# On the Inertial Range Bounds of K-41-like Magnetohydrodynamics Turbulence

**DOI:** 10.3390/e24060833

**Published:** 2022-06-16

**Authors:** Tesfalem Abate Tegegn

**Affiliations:** Department of Mathematics and Applied Mathematics, Sefako Makgatho Health Sciences University, Ga-Rankuwa, Pretoria 0204, South Africa; tesfalem.tegegn@smu.ac.za; Tel.: +27-843517105

**Keywords:** magnetohydrodynamics turbulence, harmonic analysis, Kolmogorov theory, inertial range bound, −5/3 law, 76F60, 76F02, 35M30, 76W05

## Abstract

The spectral slope of magnetohydrodynamic (MHD) turbulence varies depending on the spectral theory considered; −3/2 is the spectral slope in Kraichnan–Iroshnikov–Dobrowolny (KID) theory, −5/3 in Marsch–Matthaeus–Zhou and Goldreich–Sridhar theories, also called Kolmogorov-like (K-41-like) MHD theory, the combination of the −5/3 and −3/2 scales in Biskamp, and so on. A rigorous mathematical proof to any of these spectral theories is of great scientific interest. Motivated by the 2012 work of A. Biryuk and W. Craig (Physica D 241(2012) 426–438), we establish inertial range bounds for K-41-like phenomenon in MHD turbulent flow through a mathematical rigor; a range of wave numbers in which the spectral slope of MHD turbulence is proportional to −5/3 is established and the upper and lower bounds of this range are explicitly formulated. We also have shown that the Leray weak solution of the standard MHD model is bonded in the Fourier space, the spectral energy of the system is bounded and its average over time decreases in time.

## 1. Introduction

At a high Reynolds number fluid and plasma flows exhibit a complex random behavior called turbulence. Turbulence is observed in a great majority of fluids both in nature such as the atmosphere, river currents, oceans, solar wind, and interstitial bodies and in technical devices, such as laboratory installations, nuclear power plants, etc. Its importance in industry and physical sciences, such as making predictions about heat transfer in nuclear power plants, drag in oil pipelines, and the weather is tremendous. Besides these real-life relevant issues, the study of turbulence can assist mathematical researchers in understanding some aspects, such as the regularity of Euler’s equation, the Navier–Stokes equation, magnetohydrodynamics equations, and so on, see for instance [1].

The literature shows that the phenomenon of turbulence has captured the attention of humankind for centuries, see for instance [2]. The discovery of the Euler equations in the mid-18th century and Navier–Stokes equations in the first half of the 19th century are the major scientific and mathematical breakthroughs. Towards the end of 19th century Osborne Reynolds laid a foundation for the theory of turbulence, see [3,4], ([5], p. 488) and [6]. Reynolds number, a widely used criteria to classify whether a given flow is turbulent or not, and Reynolds averaged Navier–Stokes equations (RANS) are due to O. Reynolds. RANS is formulated by decomposing the velocity field u(x,t) in to average velocity $\bar{u}\left(x,t\right)$ over a time interval and fluctuation velocity $u^{\prime }\left(x,t\right)=u\left(x,t\right)-\bar{u}\left(x,t\right)$, and finally rewriting the Navier–Stokes equations in terms of the average velocity $\bar{u}$. In fact, RANS is still one of the most widely used models to study turbulence in fluids, see [7,8] and the references there.

The 1941 phenomenological theory of turbulence by A.N. Kolmogorov, published in a series works [9,10,11,12] postulated that the spectral energy of a fully developed turbulence decays according to the rule

(1) $$\begin{array}{c} C_{0}\epsilon^{2/3}k^{-5/3}, \end{array}$$

over a range of wavenumbers, $k\in [k_{1},k_{2}]$, also called the inertial range; where ϵ is the energy dissipation rate and $C_{0}$ is a universal constant called Kolmogorov constant. The exponents in (1) are determined by dimensional analysis. The theory is often referred to as K-41 theory or Kolmogorov’s −5/3 law. The state-of-the-art exposition of Kolmogorov’s school of turbulence can be found in the seminal monographs of Monin and Yaglom [13,14].

By the middle of the 20th century works particularly focus on MHD spectral theory started to emerge. From the earliest of such works Kraichnan [15,16] and Iroshnikov [17] can be mentioned. Unlike Kolmogorov, where the spectral energy decays proportional to $k^{-5/3}$, Kraichnan and Iroshnikov concluded that the spectral energy of a fully developed MHD turbulent flow decays proportional to $k^{-3/2}$, which later on was supported by M. Dobrowolny, A. Mangeney, and P. Veltri in [18]. Mahendra K. Verma in his review [19] said these works are the first to establish phenomenological theory on MHD turbulence, where he referred it as the Kraichnan–Iroshnikov–Dobrowolny (KID) phenomenon.

It is important to note that MHD turbulence, unlike hydrodynamic turbulence, is controlled by a combined effect of the magnetic field and the fluid velocity, see for instance [20]. Despite the difference in the formation of hydrodynamic and MHD turbulence, several authors have argued that under certain conditions the spectral energy of MHD turbulence also decays proportional to $k^{-5/3},$ which is widely accepted as a spectral slope for hydrodynamic turbulence. For instance, Marsch and Tu in [21] and Marsch in [22] suggested that the decay rate of an isotropic turbulence in sthe olar wind is very likely to be −5/3 than −3/2. Matthaeus and Zhou in [23] proposed that the larger wavenumbers (relative to the mean magnetic field) would follow the −3/2 law whereas the smaller wavenumbers would follow the −5/3 law. Biskamp in [24] proposed three different rates; −5/3 for the general MHD turbulence when Alfvén effect is neglected, −5/4 when Alfvén effects are included and the mean magnetic field is constant, and −3/2 when Alfvén effects are considered and the mean magnetic field is fluctuating. Boldyrev in [25] also concluded that MHD turbulence is not completely described by either the −3/2 or −5/3 scales; the scales depend on the strength of the external magnetic field: −3/2 scale applies when the mean magnetic field is strong while −5/3 scale applies when the external magnetic field is weak. We refer to the review by Verma [19] for the several phenomenological theories on MHD turbulence, the book by Davidson et al. [2] for the biographies and works of some of the prominent contributors to the area, and [26,27,28,29] and the references in there for interesting applications and recent developments.

The purpose of this paper is to establish a spectral range for K-41-like MHD phenomenon through mathematical rigor. The work was motivated by the 2012 paper of Andrei Biryuk and Walter Craig [30] where they established an estimate for the Leray weak solution of Navier–Stokes equations in the norm $∥\hat{\partial_{x}u\left(\cdot ,t\right)}{∥}_{L^{\infty }}$ which lead to proving the solution’s ability to satisfy Kolmogorov’s spectral law (1). J. Leray formulated weak solutions in the first half of the 1930s and considered them as turbulent solutions, see [31,32]. Following Leray’s work several authors treated weak solutions for fluid dynamic models as turbulent solution, see for instance [33,34,35,36]. Therefore, it is not surprising to see the Leray weak solution of Navier–Stokes equations obeying K-41. In a similar passion, we consider the weak solution for a system of MHD equations as a turbulent solution and attempt to show that it obeys the −5/3 spectral law over a range of wave numbers when certain conditions are met.

The dynamics of MHD flows in general is described by a system of partial differential equations given by

(2) $$\begin{array}{c} \left\{\begin{array}{cc} \partial_{t}u+\left(u\cdot \nabla \right)u+\nabla \pi -\left(b\cdot \nabla \right)b-\nu \Delta u=f_{1}, & (0,\infty )\times D,\\ \partial_{t}b+\left(u\cdot \nabla \right)b-\left(b\cdot \nabla \right)u-\eta \Delta b=f_{2}, & (0,\infty )\times D,\\ \mathrm{div}\;u=\mathrm{div}b=0, & D,\\ {u|}_{t=0}=u_{0}{,\;b|}_{t=0}=b_{0}, & D, \end{array}\right. \end{array}$$

where u=u(x,t) is the flow velocity, b=b(x,t) is the magnetic field, $\pi =P+\frac{1}{2}{\left|b\right|}^{2}$ is the total pressure on the system with *P* representing the pressure function from the equation of motion, ν>0 is the kinetic viscosity of the fluid, η>0 is the resistivity of the fluid, and the spatial domain *D* is the Euclidean space $R^{3}$. The non-homogeneous external forces $f_{1}=f_{1}\left(x,t\right),\;f_{2}=f_{2}\left(x,t\right)$ are assumed to be divergence-free and satisfy $f_{1},f_{2}\in L_{loc}^{\infty }\left(\left[0,\infty \right);H^{-1}\left(D\right)\cap L^{2}\left(D\right)\right)$, where $L_{loc}^{\infty }$ is the space of locally bounded functions, $H^{-1}$ and $L^{2}$ are the usual Sobolev and Lebesgue spaces, respectively. The derivation of Equation (2) is done by combining the Navier–Stokes equations and the Maxwell equations in some way, see [37,38,39].

We now introduce the spectral energy function, denoted by E(k,t); the spectral energy of the MHD flow model (2) is given by the surface integral

(3) $$\begin{array}{c} E\left(k,t\right):=\int_{|\xi |=k}\left(\right|\hat{u}{\left(\xi ,t\right)|}^{2}+|\hat{b}{\left(\xi ,t\right)|}^{2})dS\left(\xi \right),\;k\in \left[0,\infty \right),\;\{|\xi |=k\}\subset D, \end{array}$$

where $\hat{u}$ and $\hat{b}$ represent the Fourier transforms of *u* and *b*, respectively.

Of great scientific interest is the question of rigorous mathematical proof of the spectral theory, K-41 or otherwise, under physically admissible conditions. Therefore, our main goal will be to set the conditions on the data and to show that the spectral energy (3) satisfies −5/3 law when such conditions are met.

Before we give a formal definition to the weak solution of (2), we introduce some function spaces and their notations as they appear in [40]. We denote by $C_{0,\sigma }^{\infty }$ the set of all divergence-free smooth functions with compact support in *D*. $L_{\sigma }^{p}$ is the closure of $C_{0,\sigma }^{\infty }$ with respect to the $L^{p}$ norm in the usual sense. For 1≤p≤∞ the space $L^{p}$ stands for the usual (vector-valued) Lebesgue space over $R^{3}$. For s∈R, we denote by $H_{\sigma }^{s}$ the closure of $C_{0,\sigma }^{\infty }$ with respect to the $H^{s}$ norm.

**Definition** **1.** *Let $\left(u_{0},b_{0}\right)\in L_{\sigma }^{2}\left(D\right)$. A vector (u,b) is said to be a weak solution to *(2)* on D×[0,∞) if it satisfies the following conditions:*

*1.*  *for any T>0 the vector function (u,b) lies in the following function space,*
$$\begin{array}{c} u,\;b\in L^{\infty }\left(\left[0,T\right);L_{\sigma }^{2}\left(D\right)\right)\cap L^{2}\left(\left[0,T\right);H_{\sigma }^{1}\left(D\right)\right). \end{array}$$
*2.*  *the pair (u,b) is a distributional solution of *(2)*; i.e., for every (Φ,Ψ) in*
$$\begin{array}{c} H^{1}\left(\left(0,T\right);H_{\sigma }^{1}\cap L^{2}\right), \end{array}$$
  *with Φ(T)=Ψ(T)=0,*
$$\begin{array}{cc} \int_{0}^{T} & \{-\left(u,\partial_{t}\Phi \right)+\nu \left(\nabla u,\nabla \Phi \right)+\left(u\cdot \nabla u,\Phi \right)-\left(b\cdot \nabla b,\Phi \right)\}dt\\ & =-\left(u_{0},\Phi \left(0\right)\right)+\int_{0}^{T}\left(f_{1},\Phi \right)dt, \end{array}$$
  *and*
$$\begin{array}{cc} \int_{0}^{T} & \{-\left(b,\partial_{t}\Psi \right)+\eta \left(\nabla b,\nabla \Psi \right)+\left(u\cdot \nabla b,\Psi \right)-\left(b\cdot \nabla u,\Psi \right)\}dt\\ & =-\left(b_{0},\Psi \left(0\right)\right)+\int_{0}^{T}\left(f_{2},\Psi \right)dt. \end{array}$$
  *Furthermore $\lim_{t\to 0^{+}}u\left(\cdot ,t\right)=u_{0}\left(\cdot \right)$ and $\lim_{t\to 0^{+}}b\left(\cdot ,t\right)=b_{0}\left(\cdot \right)$ exist in the strong $L^{2}$ sense.*
*3.*  *the following energy inequality is satisfied,*
(4) $$\begin{array}{ccc} & & \frac{1}{2}\int_{D}{\left|u\left(x,t\right)\right|}^{2}+{\left|b\left(x,t\right)\right|}^{2}dx+\min\left(\nu ,\eta \right)\int_{0}^{t}\int_{D}{\left|\nabla u\left(x,s\right)\right|}^{2}+{\left|\nabla b\left(x,s\right)\right|}^{2}dxds\\ & & -\int_{0}^{t}\int_{D}u\left(x,s\right)\cdot f_{1}\left(x,s\right)+b\left(x,s\right)\cdot f_{2}\left(x,s\right)dxds\\ & & \leq \frac{1}{2}\int_{D}|u_{0}{\left(x\right)|}^{2}+{\left|b_{0}\left(x\right)\right|}^{2}dx \end{array}$$

*for all 0<t<∞.*

The rest of the paper is divided into three main sections; Section 2, Section 3 and Section 4. In Section 2 we briefly discuss Fourier transform and its properties, rewrite Equation (2) in Fourier variables, and derive prior estimates. In Section 3 we present and prove our main results whereby we drive the bounds of the spectral energy function (3) and spectral energy bounds. Finally, Section 4 is conclusion.

## 2. Estimates for the Solution Field (u,b) in a Fourier Space

### 2.1. The Fourier Transform

The Fourier transform of an integrable function *u*, denoted by $\hat{u}$, is defined by

$$\begin{array}{c} \hat{u}\left(\xi \right)=\int_{D}e^{-i\xi \cdot x}u\left(x\right)dx. \end{array}$$

The Fourier transform has several interesting properties, among them the following three are of great importance to this work;

(5) $$\begin{array}{c} {∥u∥}_{L^{2}\left(D\right)}^{2}={∥\hat{u}∥}_{L^{2}\left(D\right)}^{2}, \end{array}$$

(6) $$\begin{array}{c} \hat{\partial_{x}^{\alpha }u\left(x\right)}={\left(i\xi \right)}^{\alpha }\partial_{\xi }^{\alpha }\hat{u}\left(\xi \right)\;\mathrm{and}\;\hat{x^{\alpha }u\left(x\right)}={\left(-i\right)}^{\alpha }\partial_{\xi }^{\alpha }\hat{u}\left(\xi \right), \end{array}$$

and

(7) $$\begin{array}{c} \hat{uv}=\hat{u}*\hat{v}\;\mathrm{and}\;\hat{u*v}=\hat{u}\;\hat{v}. \end{array}$$

In (6), $\partial_{x}^{\alpha }$ and $\partial_{\xi }^{\alpha }$ indicate the $\alpha^{th}$ order derivative with respect to space variables in the Euclidean and Fourier spaces respectively, ∗ in (7) is the convolution operator and Equation (5) is the Parseval–Plancherel identity. For the detail of these and other properties of the Fourier transform we refer to [41,42,43].

In fact, (5) implies that the energy of the system (2) in Fourier space is equal to the energy of the system in Cartesian space. To take advantage of (5) we give an equivalent formulation for (2) in Fourier space. This is done in two steps; first we eliminate the pressure term by applying the Leray projector given by (8).

(8) $$\begin{array}{c} P\cdot :=Id-\nabla \Delta^{-1}\mathrm{div}\cdot . \end{array}$$

The application of P together with the fact that the fields *u* and *v* and the non-homogeneous terms $f_{1}$ and $f_{2}$ are divergence free reduces the system (2) to

(9) $$\begin{array}{c} \left\{\begin{array}{cc} \partial_{t}u-\nu \Delta u=P\left(\left(b\cdot \nabla \right)b\right)-P\left(\left(u\cdot \nabla \right)u\right)+f_{1}, & \\ \partial_{t}b-\eta \Delta b=P\left(\left(b\cdot \nabla \right)u\right)-P\left(\left(u\cdot \nabla \right)b\right)+f_{2}, & \\ {u|}_{t=0}=\;u_{0}{\;b|}_{t=0}=\;b_{0}. \end{array}\right. \end{array}$$

Next, we take the Fourier transform of (9) to get

(10) $$\begin{array}{c} \left\{\begin{array}{cc} {\hat{u}}_{t}+\nu {\left|k\right|}^{2}\hat{u}=\hat{\left(P((b\cdot \nabla )b)\right)}-\hat{\left(P((u\cdot \nabla )u)\right)}+\hat{f_{1}}, & \\ {\hat{b}}_{t}+\eta {\left|k\right|}^{2}\hat{b}=\hat{\left(P((b\cdot \nabla )u)\right)}-\hat{\left(P((u\cdot \nabla )b)\right)}+\hat{f_{2}}, & \\ \hat{u}{{|}_{t=o}={\hat{u}}_{0},\;\hat{b}|}_{t=o}={\hat{b}}_{0}. \end{array}\right. \end{array}$$

Thus (10) is an equivalent formulation of (2) in Fourier space.

### 2.2. A Prior Estimates

This section is devoted to finding estimates in Fourier space for solutions of (2). For ease of calculations, we define an operator

(11) $$\begin{array}{c} \Pi_{\xi }:C^{3}\to C_{\xi }^{2}\;\mathrm{by}\;\Pi_{\xi }\left(z\right)=z-\left(z\cdot \xi \right)\frac{\xi }{{\left|\xi \right|}^{2}}, \end{array}$$

where $C^{3}$ the usual three dimensional complex space and

$$\begin{array}{c} C_{\xi }^{2}:=\left\{z\in C^{3}:\xi \cdot z=0\right\}. \end{array}$$

Observe that for $\xi \in C^{3}$ and *u* divergence free, we have

(12) $$\begin{array}{ccc} \pi_{\xi }\left(\hat{u}\right) & = & \hat{u}\\ \hat{\left(P((u\cdot \nabla )b)\right)} & = & i\Pi_{\xi }\left(\int_{D}\hat{u}\left(\xi -\zeta \right)\zeta \hat{b}\left(\zeta \right)d\zeta \right)=i\Pi_{\xi }\left(\int_{D}\zeta \hat{u}\left(\xi -\zeta \right)\hat{b}\left(\zeta \right)d\zeta \right). \end{array}$$

Now plugging (12) in (10) we get,

(13) $$\begin{array}{c} \left\{\begin{array}{cc} {\hat{u}}_{t}=-\nu {\left|\xi \right|}^{2}\hat{u}+i\Pi_{\xi }\left(\int_{D}\zeta \hat{b}\left(\xi -\zeta \right)\hat{b}\left(\zeta \right)d\zeta \right)-i\Pi_{\xi }\left(\int_{D}\zeta \hat{u}\left(\xi -\zeta \right)\hat{u}\left(\zeta \right)d\zeta \right)+\hat{f_{1}}, & \\ {\hat{b}}_{t}=-\eta {\left|\xi \right|}^{2}\hat{b}+i\Pi_{\xi }\left(\int_{D}\zeta \hat{b}\left(\xi -\zeta \right)\hat{u}\left(\zeta \right)d\zeta \right)-i\Pi_{\xi }\left(\int_{D}\zeta \hat{u}\left(\xi -\zeta \right)\hat{b}\left(\zeta \right)d\zeta \right)+\hat{f_{2}}, & \\ \hat{u}{{|}_{t=o}={\hat{u}}_{0},\;\hat{b}|}_{t=o}={\hat{b}}_{0}. \end{array}\right. \end{array}$$

**Remark** **1.** *Let $B_{R}\left(0\right)$ a ball in $L^{2}\left(D\right)$ of radius R. Let $\left(u_{0},b_{0}\right)\in B_{R}\left(0\right)$ and $f_{1},f_{2}\in L_{loc}^{\infty }(\left[0,\infty \right);$$H^{-1}\left(D\right)\cap L^{2}\left(D\right))$. If an appropriate frame is chosen and the total pressure *Π* is suitably normalized so that*

(14) $$\begin{array}{c} \int_{D}u\left(x,t\right)\cdot f_{1}\left(x,t\right)+b\left(x,t\right)\cdot f_{2}\left(x,t\right)dx, \end{array}$$

*is bounded, then for any T>0 there is a non negative function R(T) such that*

(15) $$\begin{array}{c} {∥u\left(\cdot ,T\right)∥}_{L^{2}}^{2}+{∥b\left(\cdot ,T\right)∥}_{L^{2}}^{2}+\min\left(\nu ,\eta \right)\int_{0}^{T}{(∥\nabla u\left(\cdot ,s\right)∥}_{L^{2}}^{2}+{∥\nabla b\left(\cdot ,s\right)∥}_{L^{2}}^{2})ds\leq R^{2}\left(T\right). \end{array}$$

*Furthermore, when $f_{1}\equiv f_{2}\equiv 0$, the bound R(T)=R is a constant fully determined by the initial data $\left(u_{0},b_{0}\right)$. In this case one could actually take R to be the right hand side (RHS) of *(4)* and $B_{R}\left(0\right)$, a ball of radius R and center 0, becomes an invariant (set A is said to be an invariant (future invariant) set with respect to a function φ or family of functions $\left\{\varphi \left(t\right):t\in \left[0,\infty \right)\right\}$, if*

φ(0)∈A⇒φ(t)∈A,∀t≥0.

*) set for the weak solution.*

Assuming that the non-homogeneous terms $f_{1}$ and $f_{2}$ are appropriately chosen so that (15) holds. With no lose of generality, one may assume from (5) that

(16) $$\begin{array}{c} ∥\hat{u}{\left(\cdot ,t\right)∥}_{L^{2}}^{2}+{∥\hat{b}\left(\cdot ,t\right)∥}_{L^{2}}^{2}\leq R^{2}\left(t\right). \end{array}$$

However, the problem is, since u,b are only distributional (weak) solutions, their Fourier transforms are not well defined at particular points, say (ξ,t), in Fourier space-time. We address the problem by taking a smooth cutoff of *u* and *b* over a cube of finite length and making use of the Paley–Wiener theorem ([42], p. 193).

Let $k\left(\neq 0\right)\in R^{3},\;0<\delta <\frac{\left|k\right|}{2\sqrt{3}}$. Define $\chi_{k}\left(\cdot \right)$ to be a smooth cutoff function of a cube $Q_{k}$ about *k* of side length 2δ such that

$$\begin{array}{c} {\hat{\chi }}_{k}\left(\xi \right)=1, \end{array}$$

on a cube of the same center with side δ and

$$\begin{array}{c} \mathrm{supp}\hat{\chi }=\{\xi \in R^{3}:\frac{\left|k\right|}{2}\leq \left|\xi \right|\leq \frac{3}{2}|k|\}. \end{array}$$

Consider the following three smooth cutoff functions defined to suit our purpose;

(17) $$\begin{array}{ccc} \left({\hat{\chi }}_{k}\left(D\right)u\right)\left(x,t\right) & := & F^{-1}\left({\hat{\chi }}_{k}\left(\xi \right)\hat{u}\left(\xi ,t\right)\right)=\left(\chi_{k}*u\right)\left(x,t\right), \end{array}$$

(18) $$\begin{array}{ccc} e_{p}\left(k,t\right) & := & {\left(\int_{D}|{\hat{\chi }}_{k}\left(\xi \right)\hat{u}{\left(\xi ,t\right)|}^{p}+{\left|{\hat{\chi }}_{k}\left(\xi \right)\hat{b}\left(\xi ,t\right)\right|}^{p}d\xi \right)}^{\frac{1}{p}}, \end{array}$$

(19) $$\begin{array}{ccc} h_{p}\left(k,t\right) & := & \underset{0\leq s\leq t}{\sup}{\left(\int_{D}\left[\right|{\hat{\chi }}_{k}\left(\xi \right)\hat{f_{1}}{\left(\xi ,t\right)|}^{p}+|{\hat{\chi }}_{k}\left(\xi \right){\hat{f}}_{1}\left(\xi ,t\right){|}^{p}]/{\left|\xi \right|}^{p}d\xi \right)}^{\frac{1}{p}}. \end{array}$$

**Remark** **2.** *Since the Fourier transform of $\chi_{k}$ is compactly supported, by Paley–Wiener theorem, ([42], Theorem 7.3.1) we have $\chi_{k}\in H^{m}$ for all m. Thus $\chi_{k}$ can be considered as a test function.*

We now have enough preparation to start working on estimating our solution in Fourier space. To establish necessary estimates, we first need to establish estimates on $e_{p},$ for p=2 followed by estimate for $e_{p}\left(k,t\right)$ for all 2≤p≤∞.

**Lemma** **1.** *Suppose that *(15)* holds and there exists a non-decreasing function $R_{1}\left(t\right)$ such that*

(20) $$\begin{array}{c} {\left(2\delta \right)}^{3/2}\sqrt{2}R^{2}\left(t\right)+2h_{2}\left(k,t\right)<\frac{\min(\nu ,\eta )}{6}R_{1}\left(t\right), \end{array}$$

*for all t∈[0,∞) and $\delta <\frac{\left|k\right|}{2\sqrt{3}}.$ If $e_{2}\left(k,0\right)<\frac{R_{1}\left(0\right)}{\left|k\right|},$ then for any t∈(0,∞) we have*

(21) $$\begin{array}{c} e_{2}\left(k,t\right)\leq \frac{R_{1}\left(t\right)}{\left|k\right|}. \end{array}$$

**Proof of** **Lemma 1.** By definition

(22) $$\begin{array}{c} e_{2}^{2}\left(k,t\right)=\int_{D}{\hat{\chi }}_{k}\left(\xi \right)\hat{u}\left(\xi ,t\right)\bar{{\hat{\chi }}_{k}\left(\xi \right)\hat{u}\left(\xi ,t\right)}+{\hat{\chi }}_{k}\left(\xi \right)\hat{b}\left(\xi ,t\right)\bar{{\hat{\chi }}_{k}\left(\xi \right)\hat{b}\left(\xi ,t\right)}d\xi . \end{array}$$

Differentiating (22) with respect to time and using Equation (13), we get

$$\begin{array}{cc} \frac{d}{dt}e_{2}^{2}\left(k,t\right)\\ = & \int_{D}\left[{\hat{\chi }}_{k}\left(\xi \right)\frac{d}{dt}\left(\hat{u}\left(\xi ,t\right)\right)\left(\bar{{\hat{\chi }}_{k}\left(\xi \right)\hat{u}\left(\xi ,t\right)}\right)+\left({\hat{\chi }}_{k}\left(\xi \right)\hat{u}\left(\xi ,t\right)\right)\bar{{\hat{\chi }}_{k}\left(\xi \right)}\frac{d}{dt}\left(\bar{\hat{u}\left(\xi ,t\right)}\right)\right.\\ & \left.+{\hat{\chi }}_{k}\left(\xi \right)\frac{d}{dt}\left(\hat{b}\left(\xi ,t\right)\right)\bar{{\hat{\chi }}_{k}\left(\xi \right)\hat{b}\left(\xi ,t\right)}+{\hat{\chi }}_{k}\left(\xi \right)\hat{b}\left(\xi ,t\right)\bar{{\hat{\chi }}_{k}\left(\xi \right)}\frac{d}{dt}\left(\bar{\hat{b}\left(\xi ,t\right)}\right)\right]d\xi \\ = & \int_{D}\left[{\hat{\chi }}_{k}\left(\xi \right)\left(-{\nu |\xi |}^{2}\hat{u}+i\Pi_{\xi }\left(\int_{D}\zeta \hat{b}\left(\xi -\zeta \right)\hat{b}\left(\zeta \right)d\zeta \right)-\right.\right.\\ & \left.i\Pi_{\xi }\left(\int_{D}\zeta \hat{u}\left(\xi -\zeta \right)\hat{u}\left(\zeta \right)d\zeta \right)+\hat{f_{1}}\right)\left(\bar{{\hat{\chi }}_{k}\left(\xi \right)\hat{u}\left(\xi ,t\right)}\right)\\ & +\left({\hat{\chi }}_{k}\left(\xi \right)\hat{u}\left(\xi ,t\right)\right)\bar{{\hat{\chi }}_{k}\left(\xi \right)\left(-{\nu |\xi |}^{2}\hat{u}+i\Pi_{\xi }\left(\int_{D}\zeta \hat{b}\left(\xi -\zeta \right)\hat{b}\left(\zeta \right)d\zeta \right)\right.}\\ & \bar{\left.-i\Pi_{\xi }\left(\int_{D}\zeta \hat{u}\left(\xi -\zeta \right)\hat{u}\left(\zeta \right)d\zeta \right)+\hat{f_{1}}\right)}\\ & +{\hat{\chi }}_{k}\left(\xi \right)\left(-{\eta |\xi |}^{2}\hat{b}+i\Pi_{\xi }\left(\int_{D}\zeta \hat{b}\left(\xi -\zeta \right)\hat{u}\left(\zeta \right)d\zeta \right)\right.\\ & \left.-i\Pi_{\xi }\left(\int_{D}\zeta \hat{u}\left(\xi -\zeta \right)\hat{b}\left(\zeta \right)d\zeta \right)+\hat{f_{2}}\right)\bar{{\hat{\chi }}_{k}\left(\xi \right)\hat{b}\left(\xi ,t\right)}\\ & \left.+{\hat{\chi }}_{k}\left(\xi \right)\hat{b}\left(\xi ,t\right)\bar{{\hat{\chi }}_{k}\left(\xi \right)-\eta {\left|\xi \right|}^{2}\hat{b}+i\Pi_{\xi }\left(\int_{D}\zeta \hat{b}\left(\xi -\zeta \right)\hat{u}\left(\zeta \right)d\zeta \right)d\xi .}\right.\\ & \left.-\bar{i\Pi_{\xi }\left(\int_{D}\zeta \hat{u}\left(\xi -\zeta \right)\hat{b}\left(\zeta \right)d\zeta \right)+\hat{f_{2}}}\right]. \end{array}$$

Applying elementary properties of complex numbers, it follows that

(23) $$\begin{array}{cc} \frac{1}{2}\frac{d}{dt}e_{2}^{2}\left(k,t\right)\\ = & -\nu \int_{D}{\left|\xi \right|}^{2}|{\hat{\chi }}_{k}\left(\xi \right)\hat{u}{\left(\xi ,t\right)|}^{2}d\xi -\eta \int_{D}{\left|\xi \right|}^{2}{\left|{\hat{\chi }}_{k}\left(\xi \right)\hat{b}\left(\xi ,t\right)\right|}^{2}d\xi \\ & +\int_{D}ℜ\left(i\Pi_{\xi }\left(\int_{D}\hat{u}\left(\xi -\zeta \right)\cdot \zeta \hat{u}\left(\zeta \right)d\zeta \right)\bar{{\hat{\chi }}_{k}\left(\xi \right)\hat{u}\left(\xi ,t\right)}\right)d\xi \\ & +\int_{D}ℜ\left(i\Pi_{\xi }\left(\int_{D}\hat{b}\left(\xi -\zeta \right)\cdot \zeta \hat{u}\left(\zeta \right)d\zeta \right)\bar{{\hat{\chi }}_{k}\left(\xi \right)\hat{b}\left(\xi ,t\right)}\right)d\xi \\ & -\int_{D}ℜ\left(i\Pi_{\xi }\left(\int_{D}\hat{b}\left(\xi -\zeta \right)\cdot \zeta \hat{b}\left(\zeta \right)d\zeta \right)\bar{{\hat{\chi }}_{k}\left(\xi \right)\hat{u}\left(\xi ,t\right)}\right)d\xi \\ & -\int_{D}ℜ\left(i\Pi_{\xi }\left(\int_{D}\hat{u}\left(\xi -\zeta \right)\cdot \zeta \hat{b}\left(\zeta \right)d\zeta \right)\bar{{\hat{\chi }}_{k}\left(\xi \right)\hat{b}\left(\xi ,t\right)}\right)d\xi \\ & +ℜ\int_{D}{\hat{\chi }}_{k}\left(\xi \right){\hat{f}}_{1}\left(\xi ,t\right)\bar{{\hat{\chi }}_{k}\left(\xi \right)\hat{u}\left(\xi ,t\right)}d\xi +ℜ\int_{D}{\hat{\chi }}_{k}\left(\xi \right){\hat{f}}_{2}\left(\xi ,t\right)\bar{{\hat{\chi }}_{k}\left(\xi \right)\hat{b}\left(\xi ,t\right)}d\xi \\ := & I_{1}+I_{2}+I_{3}+I_{4}+I_{5}+I_{6}+I_{7}+I_{8}. \end{array}$$

For ease of calculations, we now deal with the terms on RHS of (23) separately.

(24) $$\begin{array}{cc} I_{1}+I_{2} & =-\nu \int_{D}{\left|\xi \right|}^{2}|{\hat{\chi }}_{k}\left(\xi \right)\hat{u}{\left(\xi ,t\right)|}^{2}d\xi -\eta \int_{D}{\left|\xi \right|}^{2}{\left|{\hat{\chi }}_{k}\left(\xi \right)\hat{b}\left(\xi ,t\right)\right|}^{2}d\xi \\ & \leq -\min\left(\nu ,\eta \right)\frac{{\left|k\right|}^{2}}{4}\int_{D}\left(|{\hat{\chi }}_{k}\left(\xi \right)\hat{u}{\left(\xi ,t\right)|}^{2}+{\left|{\hat{\chi }}_{k}\left(\xi \right)\hat{b}\left(\xi ,t\right)\right|}^{2}\right)d\xi . \end{array}$$

In (24) we used the fact $\xi \in \mathrm{supp}{\hat{\chi }}_{k}$; that is $\frac{\left|k\right|}{2}\leq \left|\xi \right|\leq \frac{3}{2}\left|k\right|.$

$$\begin{array}{c} I_{3}=-ℑ\int_{D}\left(\Pi_{\xi }\left(\int_{D}\hat{u}\left(\xi -\zeta \right)\cdot \zeta \hat{u}\left(\zeta \right)d\zeta \right)\bar{{\hat{\chi }}_{k}\left(\xi \right)\hat{u}\left(\xi ,t\right)}\right)d\xi , \end{array}$$

which implies

(25) $$\begin{array}{ccc} |I_{3}| & \leq & \left|\int_{D}\left(\Pi_{\xi }\left(\int_{D}\hat{u}\left(\xi -\zeta \right)\cdot \zeta \hat{u}\left(\zeta \right)d\zeta \right)\bar{{\hat{\chi }}_{k}\left(\xi \right)\hat{u}\left(\xi ,t\right)}\right)d\xi \right|\\ & \leq & ∥{\hat{\chi }}_{k}\hat{u}{\left(\cdot ,t\right)∥}_{L^{2}}{∥{\hat{\chi }}_{k}\Pi_{\xi }\left(\xi \cdot \int_{D}\hat{u}\left(\xi -\zeta \right)\hat{u}\left(\zeta \right)d\zeta \right)∥}_{L^{2}}\\ & \leq & ∥{\hat{\chi }}_{k}\hat{u}{\left(\cdot ,t\right)∥}_{L^{2}}∥\xi {\hat{\chi }}_{k}{∥}_{L^{2}}{∥\hat{u}\left(\cdot ,t\right)∥}_{L^{2}}^{2}. \end{array}$$

The estimate in (25) is due to the fact that uandb are divergence free and elementary properties of complex numbers. Hölder’s and Young’s inequalities are also used.

We know from construction of $\chi_{k}$ and Hölder’s inequality that

(26) $$\begin{array}{ccc} ∥\xi {\hat{\chi }}_{k}{∥}_{L^{2}} & \leq & {∥\xi ∥}_{L^{4}}{∥\chi_{k}∥}_{L^{4}}\\ & = & {\left(\int_{Q_{k}}{\left|\xi \right|}^{4}d\xi \right)}^{\frac{1}{4}}{\left(\int_{Q_{k}}{\left|\chi_{k}\right|}^{4}d\xi \right)}^{\frac{1}{4}}\\ & \leq & \frac{3}{2}{|k|\left(2\delta \right)}^{\frac{3}{4}}{\left(2\delta \right)}^{\frac{3}{4}}=\frac{3}{2}\left|k\right|{\left(2\delta \right)}^{\frac{3}{2}}. \end{array}$$

Thus, combining (25) and (26) we get,

(27) $$\begin{array}{c} |I_{3}|\leq \frac{3}{2}{|k|\left(2\delta \right)}^{\frac{3}{2}}∥{\hat{\chi }}_{k}\hat{u}{\left(\cdot ,t\right)∥}_{L^{2}}{∥\hat{u}\left(\cdot ,t\right)∥}_{L^{2}}^{2}. \end{array}$$

Proceeding similarly with $I_{4},\;I_{5}$ and $I_{6}$ we get

(28) $$\begin{array}{ccc} |I_{4}| & \leq & \frac{3}{2}{|k|\left(2\delta \right)}^{\frac{3}{2}}∥{\hat{\chi }}_{k}\hat{b}{\left(\cdot ,t\right)∥}_{L^{2}}∥\hat{u}{\left(\cdot ,t\right)∥}_{L^{2}}{∥\hat{b}\left(\cdot ,t\right)∥}_{L^{2}}, \end{array}$$

(29) $$\begin{array}{ccc} |I_{5}| & \leq & \frac{3}{2}{|k|\left(2\delta \right)}^{\frac{3}{2}}∥{\hat{\chi }}_{k}\hat{u}{\left(\cdot ,t\right)∥}_{L^{2}}{∥\hat{b}\left(\cdot ,t\right)∥}_{L^{2}}^{2}, \end{array}$$

(30) $$\begin{array}{ccc} |I_{6}| & \leq & \frac{3}{2}{|k|\left(2\delta \right)}^{\frac{3}{2}}∥{\hat{\chi }}_{k}\hat{b}{\left(\cdot ,t\right)∥}_{L^{2}}∥\hat{u}{\left(\cdot ,t\right)∥}_{L^{2}}{∥\hat{b}\left(\cdot ,t\right)∥}_{L^{2}}. \end{array}$$

Thanks to Hölder’s inequality, the integral $I_{7}$ is estimated as follows;

(31) $$\begin{array}{ccc} |I_{7}| & = & \left|ℜ\int_{D}{\hat{\chi }}_{k}\left(\xi \right){\hat{f}}_{1}\left(\xi ,t\right)\bar{{\hat{\chi }}_{k}\left(\xi \right)\hat{u}\left(\xi ,t\right)}d\xi \right|\\ & \leq & \left|\int_{D}\bar{{\hat{\chi }}_{k}\left(\xi \right)\hat{u}\left(\xi ,t\right)}{\hat{\chi }}_{k}\left(\xi \right)\left|\xi \right|{\hat{f}}_{1}\left(\xi ,t\right)/\left|\xi \right|d\xi \right|\\ & \leq & ∥|\xi |{\hat{\chi }}_{k}\hat{u}{\left(\cdot ,t\right)∥}_{L^{2}}{∥{\hat{\chi }}_{k}{\hat{f}}_{1}\left(\cdot ,t\right){\left|\xi \right|}^{-1}∥}_{L^{2}}\\ & \leq & \frac{3}{2}|k|∥{\hat{\chi }}_{k}\hat{u}{\left(\cdot ,t\right)∥}_{L^{2}}{∥{\hat{\chi }}_{k}{\hat{f}}_{1}\left(\cdot ,t\right){\left|\xi \right|}^{-1}∥}_{L^{2}}. \end{array}$$

Similarly, we have

(32) $$\begin{array}{ccc} |I_{8}| & \leq & \frac{3}{2}|k|∥{\hat{\chi }}_{k}\hat{b}{\left(\cdot ,t\right)∥}_{L^{2}}{∥{\hat{\chi }}_{k}{\hat{f}}_{2}\left(\cdot ,t\right){\left|\xi \right|}^{-1}∥}_{L^{2}}. \end{array}$$

Now combining the estimates (24)–(32) we obtain

(33) $$\begin{array}{cc} \frac{1}{2}\frac{d}{dt}e_{2}^{2}\left(k,t\right)\\ = & -\min\left(\nu ,\eta \right)\frac{{\left|k\right|}^{2}}{4}\int_{D}\left(|{\hat{\chi }}_{k}\left(\xi \right)\hat{u}{\left(\xi ,t\right)|}^{2}+{\left|{\hat{\chi }}_{k}\left(\xi \right)\hat{b}\left(\xi ,t\right)\right|}^{2}\right)d\xi \\ & +\frac{3}{2}{|k|\left(2\delta \right)}^{\frac{3}{2}}{∥{\hat{\chi }}_{k}\hat{u}\left(\cdot ,t\right)∥}_{L^{2}}\left(∥\hat{u}{\left(\cdot ,t\right)∥}_{L^{2}}^{2}+{∥\hat{b}\left(\cdot ,t\right)∥}_{L^{2}}^{2}\right)\\ & +{3|k|\left(2\delta \right)}^{\frac{3}{2}}∥{\hat{\chi }}_{k}\hat{b}{\left(\cdot ,t\right)∥}_{L^{2}}∥\hat{u}{\left(\cdot ,t\right)∥}_{L^{2}}{∥\hat{b}\left(\cdot ,t\right)∥}_{L^{2}}\\ & +\frac{3}{2}\left|k\right|\left[∥{\hat{\chi }}_{k}\hat{u}{\left(\cdot ,t\right)∥}_{L^{2}}∥{\hat{\chi }}_{k}{\hat{f}}_{1}\left(\cdot ,t\right){\left|\xi \right|}^{-1}{∥}_{L^{2}}+∥{\hat{\chi }}_{k}\hat{b}{\left(\cdot ,t\right)∥}_{L^{2}}{∥{\hat{\chi }}_{k}{\hat{f}}_{2}\left(\cdot ,t\right){\left|\xi \right|}^{-1}∥}_{L^{2}}\right]\\ \leq & -\min\left(\nu ,\eta \right)\frac{{\left|k\right|}^{2}}{4}e_{2}^{2}\left(k,t\right)+\frac{3}{2}\left|k\right|{\left(2\delta \right)}^{3/2}\left(∥{\hat{\chi }}_{k}\hat{u}∥+∥{\hat{\chi }}_{k}\hat{b}∥\right)\left(∥\hat{u}{∥}^{2}+{∥\hat{b}∥}^{2}\right)\\ & +\frac{3}{2}\left|k\right|\left(∥{\hat{\chi }}_{k}\hat{u}∥+∥{\hat{\chi }}_{k}\hat{b}∥\right)\left(∥{\hat{\chi }}_{k}{\hat{f}}_{1}/|\xi |∥+∥{\hat{\chi }}_{k}{\hat{f}}_{2}/|\xi |∥\right)\\ \leq & -\min\left(\nu ,\eta \right)\frac{{\left|k\right|}^{2}}{4}e_{2}^{2}\left(k,t\right)+\frac{3}{2}\left|k\right|{\left(2\delta \right)}^{3/2}\sqrt{2}e_{2}\left(k,t\right)\left(∥\hat{u}{∥}^{2}+{∥\hat{b}∥}^{2}\right)\\ & +\frac{3}{2}\left|k\right|\sqrt{2}e_{2}\left(k,t\right)\left(∥{\hat{\chi }}_{k}{\hat{f}}_{1}/|\xi |∥+∥{\hat{\chi }}_{k}{\hat{f}}_{2}/|\xi |∥\right)\\ \leq & -\min\left(\nu ,\eta \right)\frac{{\left|k\right|}^{2}}{4}e_{2}^{2}\left(k,t\right)+\frac{3}{2}\left|k\right|e_{2}\left(k,t\right)\left({\left(2\delta \right)}^{3/2}\sqrt{2}R^{2}\left(t\right)+2h_{2}\left(k,t\right)\right). \end{array}$$

Here we used Serrine’s inequality ([44], Lemma 1) to estimate upper bounds for $∥{\hat{\chi }}_{k}\hat{u}∥+∥{\hat{\chi }}_{k}\hat{b}∥$ and $∥{\hat{\chi }}_{k}{\hat{f}}_{1}/|\xi |∥+∥{\hat{\chi }}_{k}{\hat{f}}_{2}/|\xi |∥$ respectively as;

$$\begin{array}{ccc} & & ∥{\hat{\chi }}_{k}\hat{u}∥+∥{\hat{\chi }}_{k}\hat{b}∥\leq \sqrt{2}e_{2}\left(k,t\right),\\ & & ∥{\hat{\chi }}_{k}{\hat{f}}_{1}/|\xi |∥+∥{\hat{\chi }}_{k}{\hat{f}}_{2}/|\xi |∥\leq \sqrt{2}h_{2}. \end{array}$$

Now define the set $B_{R_{1}}$ by,

(34) $$\begin{array}{c} B_{R_{1}}=\left\{e:e\leq R_{1}/\left|k\right|\right\}=\left\{e\left(k,t\right):e\left(k,t\right)\leq R_{1}\left(t\right)/\left|k\right|\right\}. \end{array}$$

When $e\left(k,t\right)=e_{2}\left(k,t\right)=\frac{R_{1}\left(t\right)}{\left|k\right|}$ in (34), we get

$$\begin{array}{cc} & \frac{1}{2}\frac{d}{dt}e_{2}^{2}\left(k,t\right)<\frac{-\min(\nu ,\eta )}{4}R_{1}^{2}\left(t\right)+\frac{3}{2}R_{1}\left(t\right)\frac{\min(\nu ,\eta )}{6}R_{1}\left(t\right)\leq 0. \end{array}$$

Then by chain rule and from the fact that $e_{2}\left(k,t\right)\geq 0$, we conclude that

(35) $$\begin{array}{c} \frac{d}{dt}e_{2}\left(k,t\right)<0. \end{array}$$

Indeed, (35) implies that $B_{R_{1}}$ is an attracting set for $e_{2}\left(k,t\right)$. Therefore, if $e_{2}\left(k,0\right)<\frac{R_{1}\left(0\right)}{\left|k\right|},$ then $e_{2}\left(k,t\right)<\frac{R_{1}\left(t\right)}{\left|k\right|}$ for all t∈(0,∞). □

**Lemma** **2.** *Suppose that for a given $k\in R^{3}$ and 2≤p<∞ there is a non-decreasing function $R_{1}\left(t\right)$ that satisfies the condition*

$$\begin{array}{c} 2^{\frac{1}{p}}{\left(2\delta \right)}^{3/p}R^{2}\left(t\right)+2h_{p}\left(k,t\right)<\frac{\min(\nu ,\eta )}{6}R_{1}\left(t\right), \end{array}$$

*for $0<\delta <|k|/2\sqrt{3}$.*

*If a solution to *(2)* initially satisfies*

$$\begin{array}{c} e_{p}\left(k,0\right)<R_{1}\left(0\right)/\left|k\right|, \end{array}$$

*then for all 0<t<∞,*

$$\begin{array}{c} e_{p}\left(k,t\right)<\frac{R_{1}\left(t\right)}{\left|k\right|}. \end{array}$$

**Proof of** **Lemma 2.** The proof follows same procedure as the proof of Lemma 1. We begin by taking the time derivative of $e_{p}^{p}\left(k,t\right)$.

(36) $$\begin{array}{cc} \frac{d}{dt}e_{p}^{p}\left(k,t\right)\\ = & \partial_{t}\int {\left|{\hat{\chi }}_{k}\left(\xi \right)\hat{u}\left(\xi ,t\right)\right|}^{p}+{\left|{\hat{\chi }}_{k}\left(\xi \right)\hat{b}\left(\xi ,t\right)\right|}^{p}d\xi \\ = & ℜ\left\{\int \left(p|{\hat{\chi }}_{k}\left(\xi \right)\hat{u}{\left(\xi ,t\right)|}^{p-2}\left(\left({\hat{\chi }}_{k}\left(\xi \right)\partial_{t}\hat{u}\left(\xi ,t\right)\right)\left(\bar{{\hat{\chi }}_{k}\left(\xi \right)\hat{u}\left(\xi ,t\right)}\right)\right)\right.\right.\\ & \left.\left.+p|{\hat{\chi }}_{k}\left(\xi \right)\hat{b}{\left(\xi ,t\right)|}^{p-2}\left(\left({\hat{\chi }}_{k}\left(\xi \right)\partial_{t}\hat{b}\left(\xi ,t\right)\right)\left(\bar{{\hat{\chi }}_{k}\left(\xi \right)\hat{b}\left(\xi ,t\right)}\right)\right)\right)d\xi \right\}\\ = & -\nu \int p{\left|\xi \right|}^{2}|{\hat{\chi }}_{k}\left(\xi \right)\hat{u}{\left(\xi ,t\right)|}^{p}d\xi -\eta \int p{\left|\xi \right|}^{2}{\left|{\hat{\chi }}_{k}\left(\xi \right)\hat{b}\left(\xi ,t\right)\right|}^{p}d\xi \\ & +ℜ\int ip|{\hat{\chi }}_{k}\left(\xi \right)\hat{u}{\left(\xi ,t\right)|}^{p-2}\bar{{\hat{\chi }}_{k}\left(\xi \right)\hat{u}\left(\xi ,t\right)}{\hat{\chi }}_{k}\left(\xi \right)\Pi_{\xi }\int \hat{u}\left(\xi -\zeta \right)\zeta \hat{u}\left(\zeta \right)d\zeta d\xi \\ & +ℜ\int ip|{\hat{\chi }}_{k}\left(\xi \right)\hat{u}{\left(\xi ,t\right)|}^{p-2}\bar{{\hat{\chi }}_{k}\left(\xi \right)\hat{u}\left(\xi ,t\right)}{\hat{\chi }}_{k}\left(\xi \right)\Pi_{\xi }\int \hat{b}\left(\xi -\zeta \right)\zeta \hat{b}\left(\zeta \right)d\zeta d\xi \\ & +ℜ\int ip|{\hat{\chi }}_{k}\left(\xi \right)\hat{b}{\left(\xi ,t\right)|}^{p-2}\bar{{\hat{\chi }}_{k}\left(\xi \right)\hat{b}\left(\xi ,t\right)}{\hat{\chi }}_{k}\left(\xi \right)\Pi_{\xi }\int \hat{b}\left(\xi -\zeta \right)\zeta \hat{u}\left(\zeta \right)d\zeta d\xi \\ & +ℜ\int ip|{\hat{\chi }}_{k}\left(\xi \right)\hat{b}{\left(\xi ,t\right)|}^{p-2}\bar{{\hat{\chi }}_{k}\left(\xi \right)\hat{b}\left(\xi ,t\right)}{\hat{\chi }}_{k}\left(\xi \right)\Pi_{\xi }\int \hat{u}\left(\xi -\zeta \right)\zeta \hat{b}\left(\zeta \right)d\zeta d\xi \\ & +ℜ\int p|{\hat{\chi }}_{k}\left(\xi \right)\hat{u}{\left(\xi ,t\right)|}^{p-2}\bar{{\hat{\chi }}_{k}\left(\xi \right)\hat{u}\left(\xi ,t\right)}{\hat{\chi }}_{k}\left(\xi \right){\hat{f}}_{1}\left(\xi ,t\right)d\xi \\ & +ℜ\int p|{\hat{\chi }}_{k}\left(\xi \right)\hat{b}{\left(\xi ,t\right)|}^{p-2}\bar{{\hat{\chi }}_{k}\left(\xi \right)\hat{b}\left(\xi ,t\right)}{\hat{\chi }}_{k}\left(\xi \right){\hat{f}}_{2}\left(\xi ,t\right)d\xi \\ =: & I_{1}+I_{2}+I_{3}+I_{4}+I_{5}+I_{6}+I_{7}+I_{8}. \end{array}$$

In the derivation of (36) we have used the following fact;

$$\begin{array}{ccc} \frac{d}{dt}\left|{\hat{\chi }}_{k}\left(\xi \right)\hat{b}\left(\xi ,t\right)\right| & = & \frac{d}{dt}\sqrt{{\hat{\chi }}_{k}\left(\xi \right)\hat{b}\left(\xi ,t\right)\bar{{\hat{\chi }}_{k}\left(\xi \right)\hat{b}\left(\xi ,t\right)}}\\ & = & \frac{1}{2}{\left|{\hat{\chi }}_{k}\left(\xi \right)\hat{b}\left(\xi ,t\right)\right|}^{-1}\left(\left({\hat{\chi }}_{k}\left(\xi \right)\partial_{t}\hat{b}\left(\xi ,t\right)\right)\left(\bar{{\hat{\chi }}_{k}\left(\xi \right)\hat{b}\left(\xi ,t\right)}\right)\right.\\ & & \left.+\bar{\left({\hat{\chi }}_{k}\left(\xi \right)\partial_{t}\hat{b}\left(\xi ,t\right)\right)\left(\bar{{\hat{\chi }}_{k}\left(\xi \right)\hat{b}\left(\xi ,t\right)}\right)}\right). \end{array}$$

We now estimate the integrals at the RHS of (36).

(37) $$\begin{array}{ccc} I_{1}+I_{2} & = & -\nu \int p{\left|\xi \right|}^{2}|{\hat{\chi }}_{k}\left(\xi \right)\hat{u}{\left(\xi ,t\right)|}^{p}d\xi -\eta \int p{\left|\xi \right|}^{2}{\left|{\hat{\chi }}_{k}\left(\xi \right)\hat{b}\left(\xi ,t\right)\right|}^{p}d\xi \\ & \leq & \frac{-{\nu p|k|}^{2}}{4}\int |{\hat{\chi }}_{k}\left(\xi \right)\hat{u}{\left(\xi ,t\right)|}^{p}d\xi +\frac{-{\eta p|k|}^{2}}{4}\int {\left|{\hat{\chi }}_{k}\left(\xi \right)\hat{b}\left(\xi ,t\right)\right|}^{p}d\xi \\ & \leq & -\frac{\min(\nu ,\eta )}{4}p{\left|k\right|}^{2}e_{p}^{p}\left(k,t\right). \end{array}$$

Here we used the fact that for $\xi \in \mathrm{supp}\;\hat{\chi }$, $\frac{\left|k\right|}{2}\leq \left|\xi \right|\leq \frac{3}{2}\left|k\right|$. Finally, thanks to Hölder’s and Young’s inequalities, we have

(38) $$\begin{array}{ccc} |I_{3}| & = & \left|ℑ\int p|{\hat{\chi }}_{k}\left(\xi \right)\hat{u}{\left(\xi ,t\right)|}^{p-2}\bar{{\hat{\chi }}_{k}\left(\xi \right)\hat{u}\left(\xi ,t\right)}{\hat{\chi }}_{k}\left(\xi \right)\Pi_{\xi }\xi \int \hat{u}\left(\xi -\zeta \right)\hat{u}\left(\zeta \right)d\zeta d\xi \right|\\ & \leq & \left|\int p|{\hat{\chi }}_{k}\left(\xi \right)\hat{u}{\left(\xi ,t\right)|}^{p-2}\bar{{\hat{\chi }}_{k}\left(\xi \right)\hat{u}\left(\xi ,t\right)}{\hat{\chi }}_{k}\left(\xi \right)\Pi_{\xi }\xi \int \hat{u}\left(\xi -\zeta \right)\hat{u}\left(\zeta \right)d\zeta d\xi \right|\\ & \leq & p{\left(\int {\left(|{\hat{\chi }}_{k}\left(\xi \right)\hat{u}{\left(\xi ,t\right)|}^{p-1}\right)}^{\frac{p}{p-1}}d\xi \right)}^{\frac{p-1}{p}}{\left(\int |{\hat{\chi }}_{k}\left(\xi \right)\Pi_{\xi }\xi \int \hat{u}\left(\xi -\zeta \right)\hat{u}{\left(\zeta \right)d\zeta |}^{p}d\xi \right)}^{\frac{1}{p}}\\ & \leq & p{\left(\int {\left(|{\hat{\chi }}_{k}\left(\xi \right)\hat{u}{\left(\xi ,t\right)|}^{p-1}\right)}^{\frac{p}{p-1}}d\xi \right)}^{\frac{p-1}{p}}{\left(\int |\xi {\hat{\chi }}_{k}{\left(\xi \right)|}^{p}d\xi \right)}^{\frac{1}{p}}{∥\int \hat{u}\left(\xi -\zeta \right)\hat{u}\left(\zeta \right)d\zeta ∥}_{L^{\infty }}\\ & \leq & p{\left(\int {\left(|{\hat{\chi }}_{k}\left(\xi \right)\hat{u}{\left(\xi ,t\right)|}^{p-1}\right)}^{\frac{p}{p-1}}d\xi \right)}^{\frac{p-1}{p}}{\left({\int |\xi |}^{p}{\left|{\hat{\chi }}_{k}\left(\xi \right)\right|}^{p}d\xi \right)}^{\frac{1}{p}}{\left(\int |\hat{u}{\left(\xi ,t\right)|}^{p}d\xi \right)}^{\frac{1}{p}}\\ & & \times {\left(\int |\hat{u}{\left(\xi ,t\right)|}^{\frac{p}{p-1}}d\xi \right)}^{\frac{p-1}{p}}. \end{array}$$

Following a similar approach yields,

(39) $$\begin{array}{ccc} |I_{4}| & \leq & p{\left(\int {\left(|{\hat{\chi }}_{k}\left(\xi \right)\hat{u}{\left(\xi ,t\right)|}^{p-1}\right)}^{\frac{p}{p-1}}d\xi \right)}^{\frac{p-1}{p}}{\left({\int |\xi |}^{p}{\left|{\hat{\chi }}_{k}\left(\xi \right)\right|}^{p}d\xi \right)}^{1/p}\\ & & \times {\left(\int |\hat{b}{\left(\xi ,t\right)|}^{p}d\xi \right)}^{1/p}{\left(\int |\hat{b}{\left(\xi ,t\right)|}^{\frac{p}{p-1}}d\xi \right)}^{\frac{p-1}{p}}, \end{array}$$

(40) $$\begin{array}{ccc} |I_{5}| & \leq & p{\left(\int {\left(|{\hat{\chi }}_{k}\left(\xi \right)\hat{b}{\left(\xi ,t\right)|}^{p-1}\right)}^{\frac{p}{p-1}}d\xi \right)}^{\frac{p-1}{p}}{\left({\int |\xi |}^{p}{\left|{\hat{\chi }}_{k}\left(\xi \right)\right|}^{p}d\xi \right)}^{1/p}\\ & & \times {\left(\int |\hat{b}{\left(\xi ,t\right)|}^{p}d\xi \right)}^{1/p}{\left(\int |\hat{u}{\left(\xi ,t\right)|}^{\frac{p}{p-1}}d\xi \right)}^{\frac{p-1}{p}}, \end{array}$$

(41) $$\begin{array}{ccc} |I_{6}| & \leq & p{\left(\int {\left(|{\hat{\chi }}_{k}\left(\xi \right)\hat{b}{\left(\xi ,t\right)|}^{p-1}\right)}^{\frac{p}{p-1}}d\xi \right)}^{\frac{p-1}{p}}{\left({\int |\xi |}^{p}{\left|{\hat{\chi }}_{k}\left(\xi \right)\right|}^{p}d\xi \right)}^{1/p}\\ & & \times {\left(\int |\hat{u}{\left(\xi ,t\right)|}^{p}d\xi \right)}^{1/p}{\left(\int |\hat{b}{\left(\xi ,t\right)|}^{\frac{p}{p-1}}d\xi \right)}^{\frac{p-1}{p}}. \end{array}$$

We now remain to estimate $I_{7}$ and $I_{8}$.

(42) $$\begin{array}{ccc} |I_{7}| & = & \left|ℜ\int p|{\hat{\chi }}_{k}\left(\xi \right)\hat{u}{\left(\xi ,t\right)|}^{p-2}\bar{{\hat{\chi }}_{k}\left(\xi \right)\hat{u}\left(\xi ,t\right)}{\hat{\chi }}_{k}\left(\xi \right){\hat{f}}_{1}\left(\xi ,t\right)d\xi \right|\\ & \leq & \left|\int p|{\hat{\chi }}_{k}\left(\xi \right)\hat{u}{\left(\xi ,t\right)|}^{p-2}\bar{{\hat{\chi }}_{k}\left(\xi \right)\hat{u}\left(\xi ,t\right)}{\hat{\chi }}_{k}\left(\xi \right){\hat{f}}_{1}\left(\xi ,t\right)d\xi \right|\\ & \leq & p{\left(\int |{\hat{\chi }}_{k}\left(\xi \right)\hat{u}{\left(\xi ,t\right)|}^{p}d\xi \right)}^{\frac{p-1}{p}}{\left(\int \frac{{\left|\xi \right|}^{p}{\left|{\hat{\chi }}_{k}\left(\xi \right){\hat{f}}_{1}\left(\xi ,t\right)\right|}^{p}}{{\left|\xi \right|}^{p}}d\xi \right)}^{p}\\ & \leq & \frac{3p}{2}\left|k\right|{\left(\int |{\hat{\chi }}_{k}\left(\xi \right)\hat{u}{\left(\xi ,t\right)|}^{p}d\xi \right)}^{\frac{p-1}{p}}{\left(\int \frac{|{\hat{\chi }}_{k}\left(\xi \right){\hat{f}}_{1}{\left(\xi ,t\right)|}^{p}}{{\left|\xi \right|}^{p}}d\xi \right)}^{1/p}. \end{array}$$

A similar approach yields,

(43) $$\begin{array}{c} |I_{8}|\leq \frac{3p}{2}\left|k\right|{\left(\int |{\hat{\chi }}_{k}\left(\xi \right)\hat{b}{\left(\xi ,t\right)|}^{p}d\xi \right)}^{\frac{p-1}{p}}{\left(\int \frac{|{\hat{\chi }}_{k}\left(\xi \right){\hat{f}}_{2}{\left(\xi ,t\right)|}^{p}}{{\left|\xi \right|}^{p}}d\xi \right)}^{1/p}. \end{array}$$

Now plugging the estimates (37)–(43) in (36) and rearranging the terms we get,

$$\begin{array}{ccc} \frac{d}{dt}e_{p}^{p}\left(k,t\right) & \leq & -\frac{\min(\nu ,\eta )}{4}p{\left|k\right|}^{2}e_{p}^{p}\left(k,t\right)\\ & & +{\left({\int |\xi |}^{p}{\left|{\hat{\chi }}_{k}\left(\xi \right)\right|}^{p}d\xi \right)}^{1/p}\left[{\left(\int |{\hat{\chi }}_{k}\left(\xi \right)\hat{u}{\left(\xi ,t\right)|}^{p}d\xi \right)}^{\frac{p-1}{p}}{∥\hat{u}∥}_{L^{2}}^{2}\right.\\ & & +{\left(\int |{\hat{\chi }}_{k}\left(\xi \right)\hat{u}{\left(\xi ,t\right)|}^{p}d\xi \right)}^{\frac{p-1}{p}}{∥\hat{b}∥}_{L^{2}}^{2}+\left.{\left(\int |{\hat{\chi }}_{k}\left(\xi \right)\hat{b}{\left(\xi ,t\right)|}^{p}d\xi \right)}^{\frac{p-1}{p}}∥\hat{u}{∥}_{L^{2}}{∥\hat{b}∥}_{L^{2}}\right.\\ & & \left.+{\left(\int |{\hat{\chi }}_{k}\left(\xi \right)\hat{b}{\left(\xi ,t\right)|}^{p}d\xi \right)}^{\frac{p-1}{p}}∥\hat{u}{∥}_{L^{2}}{∥\hat{b}∥}_{L^{2}}\right]\\ & & +\frac{3p}{2}\left|k\right|{\left(\int |{\hat{\chi }}_{k}\left(\xi \right)\hat{u}{\left(\xi ,t\right)|}^{p}d\xi \right)}^{\frac{p-1}{p}}{\left(\int \frac{|{\hat{\chi }}_{k}\left(\xi \right){\hat{f}}_{1}{\left(\xi ,t\right)|}^{p}}{{\left|\xi \right|}^{p}}\right)}^{1/p}\\ & & +\frac{3p}{2}\left|k\right|{\left(\int |{\hat{\chi }}_{k}\left(\xi \right)\hat{b}{\left(\xi ,t\right)|}^{p}d\xi \right)}^{\frac{p-1}{p}}{\left(\int \frac{|{\hat{\chi }}_{k}\left(\xi \right){\hat{f}}_{2}{\left(\xi ,t\right)|}^{p}}{{\left|\xi \right|}^{p}}d\xi \right)}^{1/p}. \end{array}$$

We know from the property of ${\hat{\chi }}_{k}$ that ${\left({\int |\xi |}^{p}{\left|{\hat{\chi }}_{k}\left(\xi \right)\right|}^{p}d\xi \right)}^{1/p}$ is bounded from above as

(44) $$\begin{array}{c} {\left(\int {\left|\xi \right|}^{p}{\left|{\hat{\chi }}_{k}\left(\xi \right)\right|}^{p}d\xi \right)}^{1/p}\leq \frac{3|k|}{2}{\left(2\delta \right)}^{3/p}. \end{array}$$

Furthermore, we have

(45) $$\begin{array}{l} \frac{3|k|p}{2}{\left(\int |{\hat{\chi }}_{k}\left(\xi \right)\hat{u}{\left(\xi ,t\right)|}^{p}d\xi \right)}^{\frac{p-1}{p}}{\left(\int \frac{|{\hat{\chi }}_{k}\left(\xi \right){\hat{f}}_{1}{\left(\xi ,t\right)|}^{p}}{{\left|\xi \right|}^{p}}\right)}^{1/p}\\ +\frac{3|k|p}{2}{\left(\int |{\hat{\chi }}_{k}\left(\xi \right)\hat{b}{\left(\xi ,t\right)|}^{p}d\xi \right)}^{\frac{p-1}{p}}{\left(\int \frac{|{\hat{\chi }}_{k}\left(\xi \right){\hat{f}}_{2}{\left(\xi ,t\right)|}^{p}}{{\left|\xi \right|}^{p}}d\xi \right)}^{1/p}\\ \leq \frac{3|k|p}{2}\left({\left(\int |{\hat{\chi }}_{k}\left(\xi \right)\hat{u}{\left(\xi ,t\right)|}^{p}d\xi \right)}^{\frac{p-1}{p}}+{\left(\int |{\hat{\chi }}_{k}\left(\xi \right)\hat{b}{\left(\xi ,t\right)|}^{p}d\xi \right)}^{\frac{p-1}{p}}\right)\\ \;\times \left({\left(\int \frac{|{\hat{\chi }}_{k}\left(\xi \right){\hat{f}}_{1}{\left(\xi ,t\right)|}^{p}}{{\left|\xi \right|}^{p}}\right)}^{1/p}+{\left(\int \frac{|{\hat{\chi }}_{k}\left(\xi \right){\hat{f}}_{2}{\left(\xi ,t\right)|}^{p}}{{\left|\xi \right|}^{p}}\right)}^{1/p}d\xi \right)\\ \leq \frac{3|k|p}{2}2^{\frac{1}{p}}{\left(\int |{\hat{\chi }}_{k}\left(\xi \right)\hat{u}{\left(\xi ,t\right)|}^{p}d\xi +\int {\left|{\hat{\chi }}_{k}\left(\xi \right)\hat{b}\left(\xi ,t\right)\right|}^{p}d\xi \right)}^{\frac{p-1}{p}}\\ \;\times 2^{\frac{p-1}{p}}{\left(\int \frac{|{\hat{\chi }}_{k}\left(\xi \right){\hat{f}}_{1}{\left(\xi ,t\right)|}^{p}}{{\left|\xi \right|}^{p}}+\int \frac{|{\hat{\chi }}_{k}\left(\xi \right){\hat{f}}_{2}{\left(\xi ,t\right)|}^{p}}{{\left|\xi \right|}^{p}}d\xi \right)}^{1/p}\\ \leq 2\frac{3|k|p}{2}e_{p}^{p-1}\left(k,t\right)h_{p}\left(k,t\right), \end{array}$$

and

(46) $$\begin{array}{l} \left[{\left(\int |{\hat{\chi }}_{k}\left(\xi \right)\hat{u}{\left(\xi ,t\right)|}^{p}d\xi \right)}^{\frac{p-1}{p}}∥\hat{u}{∥}_{L^{2}}^{2}+{\left(\int |{\hat{\chi }}_{k}\left(\xi \right)\hat{u}{\left(\xi ,t\right)|}^{p}d\xi \right)}^{\frac{p-1}{p}}{∥\hat{b}∥}_{L^{2}}^{2}\right.\\ +\left.{\left(\int |{\hat{\chi }}_{k}\left(\xi \right)\hat{b}{\left(\xi ,t\right)|}^{p}d\xi \right)}^{\frac{p-1}{p}}∥\hat{u}{∥}_{L^{2}}∥\hat{b}{∥}_{L^{2}}+{\left(\int |{\hat{\chi }}_{k}\left(\xi \right)\hat{b}{\left(\xi ,t\right)|}^{p}d\xi \right)}^{\frac{p-1}{p}}∥\hat{u}{∥}_{L^{2}}{∥\hat{b}∥}_{L^{2}}\right]\\ ={\left(\int |{\hat{\chi }}_{k}\left(\xi \right)\hat{u}{\left(\xi ,t\right)|}^{p}d\xi \right)}^{\frac{p-1}{p}}\left(∥\hat{u}{∥}^{2}+{∥\hat{b}∥}^{2}\right)+2{\left(\int |{\hat{\chi }}_{k}\left(\xi \right)\hat{b}{\left(\xi ,t\right)|}^{p}d\xi \right)}^{\frac{p-1}{p}}∥\hat{u}∥∥\hat{b}∥\\ \leq \left({\left(\int |{\hat{\chi }}_{k}\left(\xi \right)\hat{u}{\left(\xi ,t\right)|}^{p}d\xi \right)}^{\frac{p-1}{p}}+{\left(\int |{\hat{\chi }}_{k}\left(\xi \right)\hat{b}{\left(\xi ,t\right)|}^{p}d\xi \right)}^{\frac{p-1}{p}}\right)\left(∥\hat{u}{∥}^{2}+{∥\hat{b}∥}^{2}\right)\\ \leq \sqrt[{p}]{2}e_{p}^{p-1}\left(k,t\right)R^{2}\left(t\right). \end{array}$$

We next put (44)–(46) together to get,

$$\begin{array}{cc} \frac{d}{dt}e_{p}^{p}\left(k,t\right)\\ \leq & -\frac{\min(\nu ,\eta )}{4}p{\left|k\right|}^{2}e_{p}^{p}\left(k,t\right)+\frac{3|k|}{2}{\left(2\delta \right)}^{3/p}2^{\frac{1}{p}}e_{p}^{p-1}\left(k,t\right)R^{2}\left(t\right)+\\ & 2\frac{3|k|p}{2}e_{p}^{p-1}\left(k,t\right)h_{p}\left(k,t\right)\\ \leq & -\frac{\min(\nu ,\eta )}{4}p{\left|k\right|}^{2}e_{p}^{p}\left(k,t\right)+\frac{3|k|}{2}pe_{p}^{p-1}\left(k,t\right)\left(2^{\frac{1}{p}}{\left(2\delta \right)}^{3/p}R^{2}\left(t\right)+2h_{p}\left(k,t\right)\right). \end{array}$$

Once again we consider the set

$$\begin{array}{c} B_{R_{1}}=\left\{e\left(k,t\right):0\leq e\left(k,t\right)\leq \frac{R_{1}\left(t\right)}{\left|k\right|}\right\}. \end{array}$$

Setting $e\left(k,t\right)=e_{p}\left(k,t\right)=\frac{R_{1}\left(t\right)}{\left|k\right|}$, on the boundary such that $\left|k\right|e_{p}\left(k,t\right)=R_{1}\left(t\right)$,

(47) $$\begin{array}{ccc} \frac{d}{dt}e_{p}^{p}\left(k,t\right) & \leq & -\frac{\nu }{4}p{\left|k\right|}^{2}\frac{R_{1}^{p}\left(t\right)}{{\left|k\right|}^{p}}+p\frac{3|k|}{2}\frac{R_{1}^{p-1}\left(t\right)}{{\left|k\right|}^{p-1}}\left(2^{\frac{1}{p}}{\left(2\delta \right)}^{3/p}R^{2}\left(t\right)+2h_{p}\left(k,t\right)\right)\\ & < & -\frac{\min(\nu ,\eta )}{4}p{\left|k\right|}^{2}\frac{R_{1}^{p}\left(t\right)}{{\left|k\right|}^{p}}+p\frac{3|k|}{2}\frac{R_{1}^{p-1}\left(t\right)}{{\left|k\right|}^{p-1}}\frac{\min(\nu ,\eta )}{6}R_{1}\left(t\right)=0 \end{array}$$

Here we used the condition that $2^{\frac{1}{p}}{\left(2\delta \right)}^{3/p}R^{2}\left(t\right)+2h_{p}\left(k,t\right)<\frac{\min(\nu ,\eta )}{6}R_{1}\left(t\right)$. Thus, (47) implies $B_{R_{1}}$ is an attracting set for $e_{p}\left(k,t\right).$ Therefore, if $e_{p}\left(k,0\right)<\frac{R_{1}\left(0\right)}{\left|k\right|}$, then $e_{p}\left(k,t\right)<\frac{R_{1}\left(t\right)}{\left|k\right|}$ for all $t\in R^{+}$. □

The following two theorems are the main results of this section, which are direct consequences of Lemmas 1 and 2.

**Theorem** **1.** *Let the assumptions of Lemma 2 hold. If the weak solution (u,b) of *(2)* satisfies the initial condition*

$$\begin{array}{c} \underset{2\leq p<\infty }{\sup}e_{p}\left(k,0\right)<\frac{R_{1}\left(0\right)}{\left|k\right|}, \end{array}$$

*then for all t>0,*

$$\begin{array}{c} \underset{2\leq p<\infty }{\sup}e_{p}\left(k,t\right)<\frac{R_{1}\left(t\right)}{\left|k\right|}, \end{array}$$

*holds.*

**Theorem** **2.** *Suppose the weak solution (u,b) of *(2)* satisfies *(15)* and $\underset{2\leq p<\infty }{\sup}e_{p}\left(k,0\right)<\frac{R_{1}\left(0\right)}{\left|k\right|}$. Then for all $T\in R^{+}$, we have*

(48) $$\begin{array}{c} \int_{0}^{T}\underset{2\leq p<\infty }{\sup}e_{p}\left(k,t\right)dt\leq \frac{R_{2}^{2}\left(T\right)}{{\min\left(\nu ,\eta \right)|k|}^{4}}, \end{array}$$

*and*

(49) $$\begin{array}{c} R_{2}\left(T\right):=\frac{1}{2}\left(R_{3}\left(T\right)+\sqrt{4R_{1}^{2}\left(0\right)+R_{3}^{2}\left(T\right)}\right) \end{array}$$

*where*

(50) $$\begin{array}{ccc} R_{3}\left(T\right) & = & \frac{2R^{2}\left(T\right)}{\min(\nu ,\eta )}+\frac{2F_{\infty }\left(T\right)}{\sqrt{\min(\nu ,\eta )}},\\ F_{\infty }\left(T\right) & = & \underset{k\in R\;\{0\}}{\sup}\left(\int_{0}^{T}|{\hat{\chi }}_{k}\left(\xi \right){\hat{f}}_{1}{|}_{L_{\infty }}^{2}+{\left|{\hat{\chi }}_{k}\left(\xi \right){\hat{f}}_{2}\right|}_{L_{\infty }}^{2}dt\right) \end{array}$$

**Proof of Theorem** **1.** The proof is very direct. Lemma 2 implies that $e_{p}\left(k,t\right)$ is bounded uniformly in *p*. Then taking the supremum over all 2≤p<∞ concludes the proof. □

**Proof of Theorem** **2.** Recalling the definition of $e_{p}\left(k,t\right)$ from (18), we have

$$\begin{array}{c} e_{p}^{2}\left(k,t\right)={\left(\int |{\hat{\chi }}_{k}\left(\xi \right)\hat{u}{\left(\xi ,t\right|}^{p}+|{\hat{\chi }}_{k}\left(\xi \right)\hat{b}{\left(\xi ,t\right|}^{p}d\xi \right)}^{\frac{2}{p}}. \end{array}$$

Now taking the derivative in time,

(51) $$\begin{array}{c} \frac{\partial }{\partial_{t}}e_{p}^{2}\left(k,t\right)=\frac{2}{p}{\left(\int |{\hat{\chi }}_{k}\left(\xi \right)\hat{u}{\left(\xi ,t\right)|}^{p}+{\left|{\hat{\chi }}_{k}\left(\xi \right)\hat{b}\left(\xi ,t\right)\right|}^{p}d\xi \right)}^{\frac{2}{p}-1}\frac{\partial }{\partial_{t}}e_{p}^{p}\left(k,t\right). \end{array}$$

We now plug (36) in (51) to get,

(52) $$\begin{array}{cc} \frac{\partial }{\partial_{t}}e_{p}^{2}\left(k,t\right)\\ = & \left[\frac{2}{p}{\left(\int |{\hat{\chi }}_{k}\left(\xi \right)\hat{u}{\left(\xi ,t\right)|}^{p}+{\left|{\hat{\chi }}_{k}\left(\xi \right)\hat{b}\left(\xi ,t\right)\right|}^{p}d\xi \right)}^{\frac{2}{p}-1}\right]\\ & \times \left[-{\nu \int p|\xi |}^{2}|{\hat{\chi }}_{k}\left(\xi \right)\hat{u}{\left(\xi ,t\right)|}^{p}d\xi -{\eta \int p|\xi |}^{2}{\left|{\hat{\chi }}_{k}\left(\xi \right)\hat{b}\left(\xi ,t\right)\right|}^{p}d\xi \right.\\ & +ℜ\int ip|{\hat{\chi }}_{k}\left(\xi \right)\hat{u}{\left(\xi ,t\right)|}^{p-2}\bar{{\hat{\chi }}_{k}\left(\xi \right)\hat{u}\left(\xi ,t\right)}{\hat{\chi }}_{k}\left(\xi \right)\Pi_{\xi }\int \hat{u}\left(\xi -\zeta \right)\zeta \hat{u}\left(\zeta \right)d\zeta d\xi \\ & +ℜ\int ip|{\hat{\chi }}_{k}\left(\xi \right)\hat{u}{\left(\xi ,t\right)|}^{p-2}\bar{{\hat{\chi }}_{k}\left(\xi \right)\hat{u}\left(\xi ,t\right)}{\hat{\chi }}_{k}\left(\xi \right)\Pi_{\xi }\int \hat{b}\left(\xi -\zeta \right)\zeta \hat{b}\left(\zeta \right)d\zeta d\xi \\ & +ℜ\int ip|{\hat{\chi }}_{k}\left(\xi \right)\hat{b}{\left(\xi ,t\right)|}^{p-2}\bar{{\hat{\chi }}_{k}\left(\xi \right)\hat{b}\left(\xi ,t\right)}{\hat{\chi }}_{k}\left(\xi \right)\Pi_{\xi }\int \hat{b}\left(\xi -\zeta \right)\zeta \hat{u}\left(\zeta \right)d\zeta d\xi \\ & +ℜ\int ip|{\hat{\chi }}_{k}\left(\xi \right)\hat{b}{\left(\xi ,t\right)|}^{p-2}\bar{{\hat{\chi }}_{k}\left(\xi \right)\hat{b}\left(\xi ,t\right)}{\hat{\chi }}_{k}\left(\xi \right)\Pi_{\xi }\int \hat{u}\left(\xi -\zeta \right)\zeta \hat{b}\left(\zeta \right)d\zeta d\xi \\ & +ℜ\int p|{\hat{\chi }}_{k}\left(\xi \right)\hat{u}{\left(\xi ,t\right)|}^{p-2}\bar{{\hat{\chi }}_{k}\left(\xi \right)\hat{u}\left(\xi ,t\right)}{\hat{\chi }}_{k}\left(\xi \right)\hat{f_{1}}\left(\xi ,t\right)d\xi \\ & \left.+ℜ\int p|{\hat{\chi }}_{k}\left(\xi \right)\hat{b}{\left(\xi ,t\right)|}^{p-2}\bar{{\hat{\chi }}_{k}\left(\xi \right)\hat{b}\left(\xi ,t\right)}{\hat{\chi }}_{k}\left(\xi \right)\hat{f_{2}}\left(\xi ,t\right)d\xi \right]. \end{array}$$

For the sake of calculation simplicity, we split the RHS of (52) in to the following integrals.

$$\begin{array}{ccc} I_{1} & := & -2\nu {\left(\int |{\hat{\chi }}_{k}\left(\xi \right)\hat{u}{\left(\xi ,t\right)|}^{p}+{\left|{\hat{\chi }}_{k}\left(\xi \right)\hat{b}\left(\xi ,t\right)\right|}^{p}d\xi \right)}^{\frac{2}{p}-1}\\ & & \times \left({\int |\xi |}^{2}|{\hat{\chi }}_{k}\left(\xi \right)\hat{u}{\left(\xi ,t\right)|}^{p}+{\left|\xi \right|}^{2}{\left|{\hat{\chi }}_{k}\left(\xi \right)\hat{b}\left(\xi ,t\right)\right|}^{p}d\xi \right),\\ I_{2} & := & 2{\left(\int |{\hat{\chi }}_{k}\left(\xi \right)\hat{u}{\left(\xi ,t\right)|}^{p}+{\left|{\hat{\chi }}_{k}\left(\xi \right)\hat{b}\left(\xi ,t\right)\right|}^{p}d\xi \right)}^{\frac{2}{p}-1}\\ & & \times ℜ\int \left(i|{\hat{\chi }}_{k}\left(\xi \right)\hat{u}{\left(\xi ,t\right)|}^{p-2}\bar{{\hat{\chi }}_{k}\left(\xi \right)\hat{u}\left(\xi ,t\right)}{\hat{\chi }}_{k}\left(\xi \right)\Pi_{\xi }\int \hat{u}\left(\xi -\zeta \right)\zeta \hat{u}\left(\zeta \right)d\zeta \right)d\xi ,\\ I_{3} & := & 2{\left(\int |{\hat{\chi }}_{k}\left(\xi \right)\hat{u}{\left(\xi ,t\right)|}^{p}+{\left|{\hat{\chi }}_{k}\left(\xi \right)\hat{b}\left(\xi ,t\right)\right|}^{p}d\xi \right)}^{\frac{2}{p}-1}\\ & & \times ℜ\int \left(i|{\hat{\chi }}_{k}\left(\xi \right)\hat{u}{\left(\xi ,t\right)|}^{p-2}\bar{{\hat{\chi }}_{k}\left(\xi \right)\hat{u}\left(\xi ,t\right)}{\hat{\chi }}_{k}\left(\xi \right)\Pi_{\xi }\int \hat{b}\left(\xi -\zeta \right)\zeta \hat{b}\left(\zeta \right)d\zeta \right)d\xi ,\\ I_{4} & := & 2{\left(\int |{\hat{\chi }}_{k}\left(\xi \right)\hat{u}{\left(\xi ,t\right)|}^{p}+{\left|{\hat{\chi }}_{k}\left(\xi \right)\hat{b}\left(\xi ,t\right)\right|}^{p}d\xi \right)}^{\frac{2}{p}-1}\\ & & \times ℜ\int \left(i|{\hat{\chi }}_{k}\left(\xi \right)\hat{b}{\left(\xi ,t\right)|}^{p-2}\bar{{\hat{\chi }}_{k}\left(\xi \right)\hat{b}\left(\xi ,t\right)}{\hat{\chi }}_{k}\left(\xi \right)\Pi_{\xi }\int \hat{b}\left(\xi -\zeta \right)\zeta \hat{u}\left(\zeta \right)d\zeta \right)d\xi ,\\ I_{5} & := & 2{\left(\int |{\hat{\chi }}_{k}\left(\xi \right)\hat{u}{\left(\xi ,t\right)|}^{p}+{\left|{\hat{\chi }}_{k}\left(\xi \right)\hat{b}\left(\xi ,t\right)\right|}^{p}d\xi \right)}^{\frac{2}{p}-1}\\ & & \times ℜ\int \left(i|{\hat{\chi }}_{k}\left(\xi \right)\hat{b}{\left(\xi ,t\right)|}^{p-2}\bar{{\hat{\chi }}_{k}\left(\xi \right)\hat{b}\left(\xi ,t\right)}{\hat{\chi }}_{k}\left(\xi \right)\Pi_{\xi }\int \hat{u}\left(\xi -\zeta \right)\zeta \hat{b}\left(\zeta \right)d\zeta \right)d\xi ,\\ I_{6} & := & 2{\left(\int |{\hat{\chi }}_{k}\left(\xi \right)\hat{u}{\left(\xi ,t\right)|}^{p}+{\left|{\hat{\chi }}_{k}\left(\xi \right)\hat{b}\left(\xi ,t\right)\right|}^{p}d\xi \right)}^{\frac{2}{p}-1}\\ & & \times ℜ\int \left(|{\hat{\chi }}_{k}\left(\xi \right)\hat{u}{\left(\xi ,t\right)|}^{p-2}\bar{{\hat{\chi }}_{k}\left(\xi \right)\hat{u}\left(\xi ,t\right)}{\hat{\chi }}_{k}\left(\xi \right)\hat{f_{1}}\left(\xi ,t\right)\right)d\xi ,\\ I_{7} & := & 2{\left(\int |{\hat{\chi }}_{k}\left(\xi \right)\hat{u}{\left(\xi ,t\right)|}^{p}+{\left|{\hat{\chi }}_{k}\left(\xi \right)\hat{b}\left(\xi ,t\right)\right|}^{p}d\xi \right)}^{\frac{2}{p}-1}\\ & & \times ℜ\int \left(|{\hat{\chi }}_{k}\left(\xi \right)\hat{b}{\left(\xi ,t\right)|}^{p-2}\bar{{\hat{\chi }}_{k}\left(\xi \right)\hat{b}\left(\xi ,t\right)}{\hat{\chi }}_{k}\left(\xi \right)\hat{f_{2}}\left(\xi ,t\right)\right)d\xi . \end{array}$$

We now proceed to estimating each of these integrals $\left(I_{1}\right)–\left(I_{7}\right)$.

(53) $$\begin{array}{ccc} I_{1} & = & -2\nu {\left(\int |{\hat{\chi }}_{k}\left(\xi \right)\hat{u}{\left(\xi ,t\right)|}^{p}+{\left|{\hat{\chi }}_{k}\left(\xi \right)\hat{b}\left(\xi ,t\right)\right|}^{p}d\xi \right)}^{\frac{2}{p}-1}r\\ & & {\left({\int |\xi |}^{2}|{\hat{\chi }}_{k}\left(\xi \right)\hat{u}{\left(\xi ,t\right)|}^{p}+{\left|\xi \right|}^{2}{\left|{\hat{\chi }}_{k}\left(\xi \right)\hat{b}\left(\xi ,t\right)\right|}^{p}d\xi \right)}^{\frac{2}{p}}\\ & = & -2\nu {\left(\frac{\int |{\hat{\chi }}_{k}\left(\xi \right)\hat{u}{\left(\xi ,t\right)|}^{p}+{\left|{\hat{\chi }}_{k}\left(\xi \right)\hat{b}\left(\xi ,t\right)\right|}^{p}d\xi }{{\int |\xi |}^{2}\left(\right|{\hat{\chi }}_{k}\left(\xi \right)\hat{u}{\left(\xi ,t\right)|}^{p}+|{\hat{\chi }}_{k}\left(\xi \right)\hat{b}\left(\xi ,t\right){|}^{p})d\xi }\right)}^{\frac{2}{p}-1}\\ & & {\left({\int |\xi |}^{2}\left(\right|{\hat{\chi }}_{k}\left(\xi \right)\hat{u}{\left(\xi ,t\right)|}^{p}+|{\hat{\chi }}_{k}\left(\xi \right)\hat{b}\left(\xi ,t\right){|}^{p})d\xi \right)}^{2/p}. \end{array}$$

(54) $$\begin{array}{ccc} |I_{2}| & = & \left|2{\left(\int |{\hat{\chi }}_{k}\left(\xi \right)\hat{u}{\left(\xi ,t\right)|}^{p}+{\left|{\hat{\chi }}_{k}\left(\xi \right)\hat{b}\left(\xi ,t\right)\right|}^{p}d\xi \right)}^{\frac{2}{p}-1}\right.\\ & & \times \left.\int \left(i|{\hat{\chi }}_{k}\left(\xi \right)\hat{u}{\left(\xi ,t\right)|}^{p-2}\bar{{\hat{\chi }}_{k}\left(\xi \right)\hat{u}\left(\xi ,t\right)}{\hat{\chi }}_{k}\left(\xi \right)\Pi_{\xi }\int \hat{u}\left(\xi -\zeta \right)\zeta \hat{u}\left(\zeta \right)d\zeta \right)d\xi \right|\\ & \leq & 2{\left(\int |{\hat{\chi }}_{k}\left(\xi \right)\hat{u}{\left(\xi ,t\right)|}^{p}+{\left|{\hat{\chi }}_{k}\left(\xi \right)\hat{b}\left(\xi ,t\right)\right|}^{p}d\xi \right)}^{\frac{2}{p}-1}{\left(\int |{\hat{\chi }}_{k}\left(\xi \right)\hat{u}{\left(\xi ,t\right)|}^{p}d\xi \right)}^{\frac{p-2}{p}}\\ & & \times {\left(\int |{\hat{\chi }}_{k}\left(\xi \right)\hat{u}{\left(\xi ,t\right)|}^{p}d\xi \right)}^{\frac{1}{p}}{\left(\int |{\hat{\chi }}_{k}{\left(\xi \right)|}^{p}d\xi \right)}^{\frac{1}{p}}{∥\Pi_{\xi }\int \hat{u}\left(\xi -\zeta \right)\zeta \hat{u}\left(\zeta \right)d\zeta ∥}_{L^{\infty }}. \end{array}$$

Here we repeatedly used Hölder’s inequality. Similar calculations give us

(55) $$\begin{array}{ccc} |I_{3}| & \leq & 2{\left(\int |{\hat{\chi }}_{k}\left(\xi \right)\hat{u}{\left(\xi ,t\right)|}^{p}+{\left|{\hat{\chi }}_{k}\left(\xi \right)\hat{b}\left(\xi ,t\right)\right|}^{p}d\xi \right)}^{\frac{2}{p}-1}{\left(\int |{\hat{\chi }}_{k}\left(\xi \right)\hat{u}{\left(\xi ,t\right)|}^{p}d\xi \right)}^{\frac{p-2}{p}}\\ & & \times {\left(\int |{\hat{\chi }}_{k}\left(\xi \right)\hat{u}{\left(\xi ,t\right)|}^{p}d\xi \right)}^{\frac{1}{p}}{\left(\int |{\hat{\chi }}_{k}{\left(\xi \right)|}^{p}d\xi \right)}^{\frac{1}{p}}{∥\Pi_{\xi }\int \hat{b}\left(\xi -\zeta \right)\zeta \hat{b}\left(\zeta \right)d\zeta ∥}_{L^{\infty }}, \end{array}$$

(56) $$\begin{array}{ccc} |I_{4}| & \leq & 2{\left(\int |{\hat{\chi }}_{k}\left(\xi \right)\hat{u}{\left(\xi ,t\right)|}^{p}+{\left|{\hat{\chi }}_{k}\left(\xi \right)\hat{b}\left(\xi ,t\right)\right|}^{p}d\xi \right)}^{\frac{2}{p}-1}{\left(\int |{\hat{\chi }}_{k}\left(\xi \right)\hat{b}{\left(\xi ,t\right)|}^{p}d\xi \right)}^{\frac{p-2}{p}}\\ & & \times {\left(\int |{\hat{\chi }}_{k}\left(\xi \right)\hat{b}{\left(\xi ,t\right)|}^{p}d\xi \right)}^{\frac{1}{p}}{\left(\int |{\hat{\chi }}_{k}{\left(\xi \right)|}^{p}d\xi \right)}^{\frac{1}{p}}{∥\Pi_{\xi }\int \hat{b}\left(\xi -\zeta \right)\zeta \hat{u}\left(\zeta \right)d\zeta ∥}_{L^{\infty }}, \end{array}$$

(57) $$\begin{array}{ccc} |I_{5}| & \leq & 2{\left(\int |{\hat{\chi }}_{k}\left(\xi \right)\hat{u}{\left(\xi ,t\right)|}^{p}+{\left|{\hat{\chi }}_{k}\left(\xi \right)\hat{b}\left(\xi ,t\right)\right|}^{p}d\xi \right)}^{\frac{2}{p}-1}{\left(\int |{\hat{\chi }}_{k}\left(\xi \right)\hat{b}{\left(\xi ,t\right)|}^{p}d\xi \right)}^{\frac{p-2}{p}}\\ & & \times {\left(\int |{\hat{\chi }}_{k}\left(\xi \right)\hat{b}{\left(\xi ,t\right)|}^{p}d\xi \right)}^{\frac{1}{p}}{\left(\int |{\hat{\chi }}_{k}{\left(\xi \right)|}^{p}d\xi \right)}^{\frac{1}{p}}{∥\Pi_{\xi }\int \hat{u}\left(\xi -\zeta \right)\zeta \hat{b}\left(\zeta \right)d\zeta ∥}_{L^{\infty }}. \end{array}$$

For integrals involving the non-homogeneous forces,

(58) $$\begin{array}{ccc} |I_{6}| & \leq & 2{\left(\int |{\hat{\chi }}_{k}\left(\xi \right)\hat{u}{\left(\xi ,t\right)|}^{p}+{\left|{\hat{\chi }}_{k}\left(\xi \right)\hat{b}\left(\xi ,t\right)\right|}^{p}d\xi \right)}^{\frac{2}{p}-1}\\ & & \times \left|\int |{\hat{\chi }}_{k}\left(\xi \right)\hat{u}{\left(\xi ,t\right)|}^{p-2}\bar{{\hat{\chi }}_{k}\left(\xi \right)\hat{u}\left(\xi ,t\right)}{\hat{\chi }}_{k}\left(\xi \right){\hat{f}}_{1}\left(\xi ,t\right)d\xi \right|\\ & \leq & 2{\left(\int |{\hat{\chi }}_{k}\left(\xi \right)\hat{u}{\left(\xi ,t\right)|}^{p}+{\left|{\hat{\chi }}_{k}\left(\xi \right)\hat{b}\left(\xi ,t\right)\right|}^{p}d\xi \right)}^{\frac{2}{p}-1}{\left(\int |{\hat{\chi }}_{k}\left(\xi \right)\hat{u}{\left(\xi ,t\right)|}^{p}d\xi \right)}^{\frac{p-2}{p}}\\ & & \times {\left(\int |{\hat{\chi }}_{k}\left(\xi \right)\hat{u}{\left(\xi ,t\right)|}^{p}d\xi \right)}^{\frac{1}{p}}{\left(\int |{\hat{\chi }}_{k}\left(\xi \right){\hat{f}}_{1}{\left(\xi ,t\right)|}^{p}d\xi \right)}^{\frac{1}{p}}. \end{array}$$

Similarly,

(59) $$\begin{array}{ccc} |I_{7}| & \leq & 2{\left(\int |{\hat{\chi }}_{k}\left(\xi \right)\hat{u}{\left(\xi ,t\right)|}^{p}+{\left|{\hat{\chi }}_{k}\left(\xi \right)\hat{b}\left(\xi ,t\right)\right|}^{p}d\xi \right)}^{\frac{2}{p}-1}{\left(\int |{\hat{\chi }}_{k}\left(\xi \right)\hat{b}{\left(\xi ,t\right)|}^{p}d\xi \right)}^{\frac{p-2}{p}}\\ & & \times {\left(\int |{\hat{\chi }}_{k}\left(\xi \right)\hat{b}{\left(\xi ,t\right)|}^{p}d\xi \right)}^{\frac{1}{p}}{\left(\int |{\hat{\chi }}_{k}\left(\xi \right){\hat{f}}_{2}{\left(\xi ,t\right)|}^{p}d\xi \right)}^{\frac{1}{p}}. \end{array}$$

Now taking the time integral of (52) over the interval [0,T] we get

$$\begin{array}{c} e_{p}^{2}\left(k,T\right)-e_{p}^{2}\left(k,0\right)=\int_{0}^{T}\sum_{j=1}^{7}I_{j}dt. \end{array}$$

Then it follows from (53) that,

(60) $$\begin{array}{l} 2\min\left(\nu ,\eta \right)\int_{0}^{T}\{{\left(\frac{{\int |\xi |}^{2}\left(\right|{\hat{\chi }}_{k}\left(\xi \right)\hat{u}{\left(\xi ,t\right)|}^{p}+|{\hat{\chi }}_{k}\left(\xi \right)\hat{b}\left(\xi ,t\right){|}^{p})d\xi }{\int |{\hat{\chi }}_{k}\left(\xi \right)\hat{u}{\left(\xi ,t\right)|}^{p}+{\left|{\hat{\chi }}_{k}\left(\xi \right)\hat{b}\left(\xi ,t\right)\right|}^{p}d\xi }\right)}^{1-\frac{2}{p}}\\ \;\;\times {\left({\int |\xi |}^{2}\left(\right|{\hat{\chi }}_{k}\left(\xi \right)\hat{u}{\left(\xi ,t\right)|}^{p}+|{\hat{\chi }}_{k}\left(\xi \right)\hat{b}\left(\xi ,t\right){|}^{p})d\xi \right)}^{2/p}\}dt\\ \;\leq e_{p}^{2}\left(k,0\right)-e_{p}^{2}\left(k,T\right)+\sum_{j=2}^{7}\int_{0}^{T}\left|I_{j}\right|dt. \end{array}$$

Once again making use of the Young’s inequality gives,

$$\begin{array}{c} ∥\Pi_{\xi }\int \hat{u}\left(\xi -\zeta \right)\zeta \hat{b}{\left(\zeta \right)d\zeta ∥}_{L^{\infty }}\leq ∥\hat{u}{\left(\cdot ,t\right)∥}_{L^{2}}{∥\xi \hat{b}\left(\cdot ,t\right)∥}_{L^{2}}. \end{array}$$

Therefore,

(61) $$\begin{array}{cc} \int_{0}^{T}\left|I_{2}\right|dt\\ \leq & 2\int_{0}^{T}\left({\left(\int |{\hat{\chi }}_{k}\left(\xi \right)\hat{u}{\left(\xi ,t\right)|}^{p}+{\left|{\hat{\chi }}_{k}\left(\xi \right)\hat{b}\left(\xi ,t\right)\right|}^{p}d\xi \right)}^{\frac{2}{p}-1}{\left(\int |{\hat{\chi }}_{k}\left(\xi \right)\hat{u}{\left(\xi ,t\right)|}^{p}d\xi \right)}^{\frac{p-2}{p}}\right.\\ & {\left(\int |{\hat{\chi }}_{k}\left(\xi \right)\hat{u}{\left(\xi ,t\right)|}^{p}d\xi \right)}^{\frac{1}{p}}\left.\;{\left(\int |{\hat{\chi }}_{k}{\left(\xi \right)|}^{p}d\xi \right)}^{\frac{1}{p}}{∥\Pi_{\xi }\int \hat{u}\left(\xi -\zeta \right)\zeta \hat{u}\left(\zeta \right)d\zeta ∥}_{L^{\infty }}\right)dt\\ & \leq 2\int_{0}^{T}\left({\left(\int |{\hat{\chi }}_{k}\left(\xi \right)\hat{u}{\left(\xi ,t\right)|}^{p}+{\left|{\hat{\chi }}_{k}\left(\xi \right)\hat{b}\left(\xi ,t\right)\right|}^{p}d\xi \right)}^{\frac{2}{p}-1}{\left(\int |{\hat{\chi }}_{k}\left(\xi \right)\hat{u}{\left(\xi ,t\right)|}^{p}d\xi \right)}^{\frac{p-1}{p}}\right.\\ & \left.\;{\left(\int |{\hat{\chi }}_{k}{\left(\xi \right)|}^{p}d\xi \right)}^{\frac{1}{p}}∥\hat{u}{\left(\cdot ,t\right)∥}_{L^{2}}{∥\xi \hat{u}\left(\cdot ,t\right)∥}_{L^{2}}\right)dt. \end{array}$$

Thus, similar computations yield,

(62) $$\begin{array}{ccc} \int_{0}^{T}\left|I_{3}\right|dt & \leq & 2\int_{0}^{T}\left({\left(\int |{\hat{\chi }}_{k}\left(\xi \right)\hat{u}{\left(\xi ,t\right)|}^{p}+{\left|{\hat{\chi }}_{k}\left(\xi \right)\hat{b}\left(\xi ,t\right)\right|}^{p}d\xi \right)}^{\frac{2}{p}-1}\right.\\ & & \left.{\left(\int |{\hat{\chi }}_{k}\left(\xi \right)\hat{u}{\left(\xi ,t\right)|}^{p}d\xi \right)}^{\frac{p-1}{p}}{\left(\int |{\hat{\chi }}_{k}{\left(\xi \right)|}^{p}d\xi \right)}^{\frac{1}{p}}∥\hat{b}{\left(\cdot ,t\right)∥}_{L^{2}}{∥\xi \hat{b}\left(\cdot ,t\right)∥}_{L^{2}}\right)dt, \end{array}$$

(63) $$\begin{array}{ccc} \int_{0}^{T}\left|I_{4}\right|dt & \leq & 2\int_{0}^{T}\left({\left(\int |{\hat{\chi }}_{k}\left(\xi \right)\hat{u}{\left(\xi ,t\right)|}^{p}+{\left|{\hat{\chi }}_{k}\left(\xi \right)\hat{b}\left(\xi ,t\right)\right|}^{p}d\xi \right)}^{\frac{2}{p}-1}\right.\\ & & \left.{\left(\int |{\hat{\chi }}_{k}\left(\xi \right)\hat{b}{\left(\xi ,t\right)|}^{p}d\xi \right)}^{\frac{p-1}{p}}{\left(\int |{\hat{\chi }}_{k}{\left(\xi \right)|}^{p}d\xi \right)}^{\frac{1}{p}}∥\hat{u}{\left(\cdot ,t\right)∥}_{L^{2}}{∥\xi \hat{b}\left(\cdot ,t\right)∥}_{L^{2}}\right)dt, \end{array}$$

(64) $$\begin{array}{ccc} \int_{0}^{T}\left|I_{5}\right|dt & \leq & 2\int_{0}^{T}\left({\left(\int |{\hat{\chi }}_{k}\left(\xi \right)\hat{u}{\left(\xi ,t\right)|}^{p}+{\left|{\hat{\chi }}_{k}\left(\xi \right)\hat{b}\left(\xi ,t\right)\right|}^{p}d\xi \right)}^{\frac{2}{p}-1}\right.\\ & & \left.{\left(\int |{\hat{\chi }}_{k}\left(\xi \right)\hat{b}{\left(\xi ,t\right)|}^{p}d\xi \right)}^{\frac{p-1}{p}}{\left(\int |{\hat{\chi }}_{k}{\left(\xi \right)|}^{p}d\xi \right)}^{\frac{1}{p}}∥\hat{b}{\left(\cdot ,t\right)∥}_{L^{2}}{∥\xi \hat{u}\left(\cdot ,t\right)∥}_{L^{2}}\right)dt, \end{array}$$

(65) $$\begin{array}{ccc} \int_{0}^{T}\left|I_{6}\right|dt & \leq & 2\int_{0}^{T}{\left(\int |{\hat{\chi }}_{k}\left(\xi \right)\hat{u}{\left(\xi ,t\right)|}^{p}+{\left|{\hat{\chi }}_{k}\left(\xi \right)\hat{b}\left(\xi ,t\right)\right|}^{p}d\xi \right)}^{\frac{2}{p}-1}\\ & & {\left(\int |{\hat{\chi }}_{k}\left(\xi \right)\hat{u}{\left(\xi ,t\right)|}^{p}d\xi \right)}^{\frac{p-1}{p}}{\left(\int |{\hat{\chi }}_{k}\left(\xi \right){\hat{f}}_{1}{\left(\xi ,t\right)|}^{p}d\xi \right)}^{\frac{1}{p}}dt, \end{array}$$

(66) $$\begin{array}{ccc} \int_{0}^{T}\left|I_{7}\right|dt & \leq & 2\int_{0}^{T}{\left(\int |{\hat{\chi }}_{k}\left(\xi \right)\hat{u}{\left(\xi ,t\right)|}^{p}+{\left|{\hat{\chi }}_{k}\left(\xi \right)\hat{b}\left(\xi ,t\right)\right|}^{p}d\xi \right)}^{\frac{2}{p}-1}\\ & & {\left(\int |{\hat{\chi }}_{k}\left(\xi \right)\hat{b}{\left(\xi ,t\right)|}^{p}d\xi \right)}^{\frac{p-1}{p}}{\left(\int |{\hat{\chi }}_{k}\left(\xi \right){\hat{f}}_{2}{\left(\xi ,t\right)|}^{p}d\xi \right)}^{\frac{1}{p}}dt. \end{array}$$

Now putting estimates (61)–(64) together we get,

(67) $$\begin{array}{l} \int_{0}^{T}|I_{2}|dt+\int_{0}^{T}|I_{3}|dt+\int_{0}^{T}|I_{4}|dt+\int_{0}^{T}\left|I_{5}\right|dt\\ \leq 2{\left(\int |{\hat{\chi }}_{k}{\left(\xi \right)|}^{p}d\xi \right)}^{\frac{1}{p}}\int_{0}^{T}\{{\left(\int |{\hat{\chi }}_{k}\left(\xi \right)\hat{u}{\left(\xi ,t\right)|}^{p}+{\left|{\hat{\chi }}_{k}\left(\xi \right)\hat{b}\left(\xi ,t\right)\right|}^{p}d\xi \right)}^{\frac{2}{p}-1}\\ \;\times \left[{\left(\int |{\hat{\chi }}_{k}\left(\xi \right)\hat{u}{\left(\xi ,t\right)|}^{p}d\xi \right)}^{\frac{p-1}{p}}\left(∥\hat{u}{\left(\cdot ,t\right)∥}_{L^{2}}∥\xi \hat{u}{\left(\cdot ,t\right)∥}_{L^{2}}+∥\hat{b}{\left(\cdot ,t\right)∥}_{L^{2}}{∥\xi \hat{b}\left(\cdot ,t\right)∥}_{L^{2}}\right)\right.\\ \;+\left.{\left(\int |{\hat{\chi }}_{k}\left(\xi \right)\hat{b}{\left(\xi ,t\right)|}^{p}\right)}^{\frac{p-1}{p}}\left(∥\hat{u}{\left(\cdot ,t\right)∥}_{L^{2}}∥\xi \hat{b}{\left(\cdot ,t\right)∥}_{L^{2}}+∥\hat{b}{\left(\cdot ,t\right)∥}_{L^{2}}{∥\xi \hat{u}\left(\cdot ,t\right)∥}_{L^{2}}\right)\right]\}dt\\ \leq 2{\left(\int |{\hat{\chi }}_{k}{\left(\xi \right)|}^{p}d\xi \right)}^{\frac{1}{p}}\int_{0}^{T}\{{\left(\int |{\hat{\chi }}_{k}\left(\xi \right)\hat{u}{\left(\xi ,t\right)|}^{p}+{\left|{\hat{\chi }}_{k}\left(\xi \right)\hat{b}\left(\xi ,t\right)\right|}^{p}d\xi \right)}^{\frac{2}{p}-1}\\ \;\times \left[{\left(\int |{\hat{\chi }}_{k}\left(\xi \right)\hat{u}{\left(\xi ,t\right)|}^{p}+{\left|{\hat{\chi }}_{k}\left(\xi \right)\hat{b}\left(\xi ,t\right)\right|}^{p}d\xi \right)}^{\frac{p-1}{p}}\right.\\ \;\left.\times \left(∥\xi \hat{u}{\left(\cdot ,t\right)∥}_{L^{2}}+{∥\xi \hat{b}\left(\cdot ,t\right)∥}_{L^{2}}\right)\left(∥\hat{u}{\left(\cdot ,t\right)∥}_{L^{2}}+{∥\hat{b}\left(\cdot ,t\right)∥}_{L^{2}}\right)\right]\}dt\\ \leq 2{\left(2\delta \right)}^{\frac{3}{p}}\int_{0}^{T}{\left(\int |{\hat{\chi }}_{k}\hat{u}{|}^{p}+{\left|{\hat{\chi }}_{k}\hat{b}\right|}^{p}d\xi \right)}^{\frac{1}{p}}\left(∥\hat{u}∥+∥\hat{b}∥\right)\left(∥\xi \hat{u}∥+∥\xi \hat{b}∥\right)dt\\ \leq 2{\left(2\delta \right)}^{\frac{3}{p}}{\left(\int_{0}^{T}{\left(\int |{\hat{\chi }}_{k}\hat{u}{|}^{p}+{\left|{\hat{\chi }}_{k}\hat{b}\right|}^{p}d\xi \right)}^{\frac{2}{p}}\right)}^{\frac{1}{2}}\underset{0\leq t\leq T}{\sup}\left(∥\hat{u}∥+∥\hat{b}∥\right)\\ \;{\left(\int_{0}^{T}{\left(∥\xi \hat{u}∥+∥\xi \hat{b}∥\right)}^{2}dt\right)}^{\frac{1}{2}}\\ \leq 2{\left(2\delta \right)}^{\frac{3}{p}}{\mathrm{supp}}_{0\leq t\leq T}\left(∥\hat{u}∥+∥\hat{b}∥\right){\left(\int_{0}^{T}{\left(\int |{\hat{\chi }}_{k}\hat{u}{|}^{p}+{\left|{\hat{\chi }}_{k}\hat{b}\right|}^{p}d\xi \right)}^{\frac{2}{p}}\right)}^{\frac{1}{2}}\\ \;{\left(\int_{0}^{T}{\left(∥\nabla u∥+∥\nabla b∥\right)}^{2}dt\right)}^{\frac{1}{2}}\\ \leq 2{\left(2\delta \right)}^{\frac{3}{p}}R^{2}\left(T\right){\left(\int_{0}^{T}{\left(\int |{\hat{\chi }}_{k}\hat{u}{|}^{p}+{\left|{\hat{\chi }}_{k}\hat{b}\right|}^{p}d\xi \right)}^{\frac{2}{p}}\right)}^{\frac{1}{2}}. \end{array}$$

From (65) and (66) we have,

(68) $$\begin{array}{l} \int_{0}^{T}|I_{6}|dt+\int_{0}^{T}\left|I_{7}\right|dt\\ \leq 2\int_{0}^{T}\left\{{\left(\int |{\hat{\chi }}_{k}\left(\xi \right)\hat{u}{\left(\xi ,t\right)|}^{p}+{\left|{\hat{\chi }}_{k}\left(\xi \right)\hat{b}\left(\xi ,t\right)\right|}^{p}d\xi \right)}^{\frac{2}{p}-1}\right.\\ \;\left[{\left(\int |{\hat{\chi }}_{k}\left(\xi \right)\hat{u}{\left(\xi ,t\right)|}^{p}d\xi \right)}^{\frac{p-1}{p}}{\left(\int |{\hat{\chi }}_{k}\left(\xi \right){\hat{f}}_{1}{\left(\xi ,t\right)|}^{p}d\xi \right)}^{\frac{1}{p}}\right.\\ \;\left.+\left.{\left(\int |{\hat{\chi }}_{k}\left(\xi \right)\hat{b}{\left(\xi ,t\right)|}^{p}d\xi \right)}^{\frac{p-1}{p}}{\left(\int |{\hat{\chi }}_{k}\left(\xi \right){\hat{f}}_{2}{\left(\xi ,t\right)|}^{p}d\xi \right)}^{\frac{1}{p}}\right]\right\}dt\\ \leq 2\int_{0}^{T}\left\{{\left(\int |{\hat{\chi }}_{k}\left(\xi \right)\hat{u}{\left(\xi ,t\right)|}^{p}+{\left|{\hat{\chi }}_{k}\left(\xi \right)\hat{b}\left(\xi ,t\right)\right|}^{p}d\xi \right)}^{\frac{2}{p}-1}\right.\\ \;\left({\left(\int |{\hat{\chi }}_{k}\left(\xi \right)\hat{u}{\left(\xi ,t\right)|}^{p}d\xi \right)}^{\frac{p-1}{p}}+{\left(\int |{\hat{\chi }}_{k}\left(\xi \right)\hat{b}{\left(\xi ,t\right)|}^{p}d\xi \right)}^{\frac{p-1}{p}}\right)\\ \;\left.\left({\left(\int |{\hat{\chi }}_{k}\left(\xi \right){\hat{f}}_{2}{\left(\xi ,t\right)|}^{p}d\xi \right)}^{\frac{1}{p}}+{\left(\int |{\hat{\chi }}_{k}\left(\xi \right){\hat{f}}_{2}{\left(\xi ,t\right)|}^{p}d\xi \right)}^{\frac{1}{p}}\right)\right\}dt\\ \leq 2\int_{0}^{T}\left\{{\left(\int |{\hat{\chi }}_{k}\left(\xi \right)\hat{u}{\left(\xi ,t\right)|}^{p}+{\left|{\hat{\chi }}_{k}\left(\xi \right)\hat{b}\left(\xi ,t\right)\right|}^{p}d\xi \right)}^{\frac{2}{p}-1}\right.\\ \;{\left(\int |{\hat{\chi }}_{k}\left(\xi \right)\hat{u}{\left(\xi ,t\right)|}^{p}+{\left|{\hat{\chi }}_{k}\left(\xi \right)\hat{b}\left(\xi ,t\right)\right|}^{p}d\xi \right)}^{\frac{p-1}{p}}\\ \;\left.{\left(\int |{\hat{\chi }}_{k}\left(\xi \right){\hat{f}}_{2}{\left(\xi ,t\right)|}^{p}+{\left|{\hat{\chi }}_{k}\left(\xi \right){\hat{f}}_{2}\left(\xi ,t\right)\right|}^{p}d\xi \right)}^{\frac{1}{p}}\right\}dt\\ \leq 2{\left(\int_{0}^{T}{\left(\int |{\hat{\chi }}_{k}\left(\xi \right)\hat{u}{\left(\xi ,t\right)|}^{p}+{\left|{\hat{\chi }}_{k}\left(\xi \right)\hat{b}\left(\xi ,t\right)\right|}^{p}d\xi \right)}^{\frac{2}{p}}dt\right)}^{\frac{1}{2}}\\ \;{\left(\int_{0}^{T}{\left(\int |{\hat{\chi }}_{k}\left(\xi \right){\hat{f}}_{1}{\left(\xi ,t\right)|}^{p}+{\left|{\hat{\chi }}_{k}\left(\xi \right){\hat{f}}_{2}\left(\xi ,t\right)\right|}^{p}d\xi \right)}^{\frac{2}{p}}dt\right)}^{\frac{1}{2}}. \end{array}$$

Therefore putting (60), (67) and (68) together and using the fact that $\left|\xi \right|\geq \frac{\left|k\right|}{2}$ in the support of ${\hat{\chi }}_{k}$ gives,

(69) $$\begin{array}{l} \frac{1}{2}\min\left(\nu ,\eta \right){\left|k\right|}^{2}\int_{0}^{T}{\left(\int |{\hat{\chi }}_{k}\left(\xi \right)\hat{u}{\left(\xi ,t\right)|}^{p}+{\left|{\hat{\chi }}_{k}\left(\xi \right)\hat{b}\left(\xi ,t\right)\right|}^{p}d\xi \right)}^{\frac{2}{p}}dt\\ \;\leq 2{\left(\int_{0}^{T}{\left(\int |{\hat{\chi }}_{k}\left(\xi \right)\hat{u}{\left(\xi ,t\right)|}^{p}+{\left|{\hat{\chi }}_{k}\left(\xi \right)\hat{b}\left(\xi ,t\right)\right|}^{p}d\xi \right)}^{\frac{2}{p}}dt\right)}^{\frac{1}{2}}\left[2{\left(2\delta \right)}^{\frac{3}{p}}R^{2}\left(t\right)+\;\right.\\ \;\;\left.{\left(\int_{0}^{T}{\left(\int |{\hat{\chi }}_{k}\left(\xi \right){\hat{f}}_{1}{\left(\xi ,t\right)|}^{p}+{\left|{\hat{\chi }}_{k}\left(\xi \right){\hat{f}}_{2}\left(\xi ,t\right)\right|}^{p}d\xi \right)}^{\frac{2}{p}}dt\right)}^{\frac{1}{2}}\right]+e_{p}^{2}\left(k,0\right)-e_{p}^{2}\left(k,T\right). \end{array}$$

Now multiplying (69) by ${\left|k\right|}^{2}$,

(70) $$\begin{array}{l} {\min\left(\nu ,\eta \right)|k|}^{4}\int_{0}^{T}{\left(\int |{\hat{\chi }}_{k}\left(\xi \right)\hat{u}{\left(\xi ,t\right)|}^{p}+{\left|{\hat{\chi }}_{k}\left(\xi \right)\hat{b}\left(\xi ,t\right)\right|}^{p}d\xi \right)}^{\frac{2}{p}}dt\\ \;\leq 4{\left(\int_{0}^{T}{\left|k\right|}^{4}{\left(\int |{\hat{\chi }}_{k}\left(\xi \right)\hat{u}{\left(\xi ,t\right)|}^{p}+{\left|{\hat{\chi }}_{k}\left(\xi \right)\hat{b}\left(\xi ,t\right)\right|}^{p}d\xi \right)}^{\frac{2}{p}}dt\right)}^{\frac{1}{2}} \end{array}$$

(71) $$\begin{array}{ccc} & & \left[{\left(2\delta \right)}^{\frac{3}{p}}R^{2}\left(t\right)+{\left(\int_{0}^{T}{\left(\int |{\hat{\chi }}_{k}\left(\xi \right){\hat{f}}_{1}{\left(\xi ,t\right)|}^{p}+{\left|{\hat{\chi }}_{k}\left(\xi \right){\hat{f}}_{2}\left(\xi ,t\right)\right|}^{p}d\xi \right)}^{\frac{2}{p}}dt\right)}^{\frac{1}{2}}\right]\\ & & +{\;|k|}^{2}\left[e_{p}^{2}\left(k,0\right)-e_{p}^{2}\left(k,T\right)\right]. \end{array}$$

Define,

(72) $$\begin{array}{ccc} I_{p}^{2}\left(k,T\right) & = & \int_{0}^{T}{\left|k\right|}^{4}{\left(\int |{\hat{\chi }}_{k}\left(\xi \right)\hat{u}{\left(\xi ,t\right)|}^{p}+{\left|{\hat{\chi }}_{k}\left(\xi \right)\hat{b}\left(\xi ,t\right)\right|}^{p}d\xi \right)}^{\frac{2}{p}}dt, \end{array}$$

(73) $$\begin{array}{ccc} F_{p}\left(T\right) & = & {\left(\int_{0}^{T}{\left(\int |{\hat{\chi }}_{k}\left(\xi \right){\hat{f}}_{1}{\left(\xi ,t\right)|}^{p}+{\left|{\hat{\chi }}_{k}\left(\xi \right){\hat{f}}_{2}\left(\xi ,t\right)\right|}^{p}d\xi \right)}^{\frac{2}{p}}dt\right)}^{\frac{1}{2}}. \end{array}$$

We now put (70)–(73) together, use the assumption $e_{p}\left(k,0\right)\leq \frac{R_{1}\left(0\right)}{\left|k\right|}$ and rearrange terms to get

(74) $$\begin{array}{c} \min\left(\nu ,\eta \right)I_{p}^{2}\left(k,T\right)-4\left[{\left(2\delta \right)}^{\frac{3}{p}}R^{2}\left(T\right)+F_{p}\left(T\right)\right]I_{p}\left(k,T\right)-R_{1}^{2}\left(0\right)\leq 0. \end{array}$$

Observe that (74) is quadratic in $I_{p}$. Solving the associated quadratic equation yields

$$\begin{array}{c} \frac{4\left[{\left(2\delta \right)}^{\frac{3}{p}}R^{2}\left(T\right)+F_{p}\left(T\right)\right]\pm \sqrt{{\left(4\left[{\left(2\delta \right)}^{\frac{3}{p}}R^{2}\left(T\right)+F_{p}\left(T\right)\right]\right)}^{2}+4\min\left(\nu ,\eta \right)R_{1}^{2}\left(0\right)}}{2\min(\nu ,\eta )}. \end{array}$$

Elementary mathematics tells us that $I_{p}\left(k,t\right)$ cannot exceed the largest positive root of the associated quadratic equation, which is

$$\begin{array}{c} \frac{4\left[{\left(2\delta \right)}^{\frac{3}{p}}R^{2}\left(T\right)+F_{p}\left(T\right)\right]+\sqrt{{\left(4\left[{\left(2\delta \right)}^{\frac{3}{p}}R^{2}\left(T\right)+F_{p}\left(T\right)\right]\right)}^{2}+4\min\left(\nu ,\eta \right)R_{1}^{2}\left(0\right)}}{2\min(\nu ,\eta )}. \end{array}$$

Now set,

$$\begin{array}{c} R_{3,p}\left(T\right):=4\left[{\left(2\delta \right)}^{\frac{3}{p}}R^{2}\left(T\right)+F_{p}\left(T\right)\right]. \end{array}$$

Letting p⟶∞ completes the proof of Theorem 2. □

## 3. Estimates on the Spectral Energy Function and Inertial Ranges

This is the section where we present and prove our main results on the spectral energy function E(k,t), defined by (3), and its inertial range bounds. The results are presented in three theorems. The first theorem ensures that the spectral energy remains bounded when the initial conditions and the non-homogeneous external forces satisfy certain conditions, such as the assumptions in Remark 1. The second theorem estimates the time average of the spectral energy; it is shown that the average is always bounded and decays over time. Finally, the third theorem gives the inertial range bounds and formulates the conditions expected from the parameters, such as the dissipation rate, the universal constant, and viscosity coefficients so that the spectral energy decays accordingly with K-41. This is done by comparing E(k,t) with Kolmogorov’s spectral function $E_{K}\left(k\right)$ given by (1), i.e.,

(75) $$\begin{array}{c} E_{K}\left(k\right)=C_{0}\epsilon^{\frac{2}{3}}k^{-\frac{5}{3}}, \end{array}$$

defined over a range of wave numbers called the inertial range; where $C_{0}$ is a universal constant called Kolmogorov constant and ϵ is the energy dissipation rate.

**Remark** **3.** *Equation *(75)* is similar to Equation (106) of ([19], p. 267) where $C_{0}$ and ϵ were referred to as Kolmogorov constants for MHD turbulence and energy flux, respectively, instead of Kolmogorov’s constant and energy dissipation rate.*

Recall that the spectral energy function for the MHD system (76)

(76) $$\begin{array}{c} \left\{\begin{array}{cc} \partial_{t}u+\left(u\cdot \nabla \right)u+\nabla \pi -\left(b\cdot \nabla \right)b-\nu \Delta u=f_{1} & (0,\infty )\times D,\\ \partial_{t}b+\left(u\cdot \nabla \right)b-\left(b\cdot \nabla \right)u-\eta \Delta b=f_{2} & (0,\infty )\times D,\\ \mathrm{div}\;u=\mathrm{div}b=0 & D,\\ {u|}_{t=0}=u_{0}{,\;b|}_{t=0}=b_{0} & D, \end{array}\right. \end{array}$$

is given by the spherical integral

(77) $$\begin{array}{c} E\left(k,t\right)=\int_{|\xi |=k}\left(\right|\hat{u}{\left(\xi ,t\right)|}^{2}+|\hat{b}\left(\xi ,t\right){|}^{2})dS\left(\xi \right), \end{array}$$

where 0≤k<∞ is a radial coordinate in Fourier space.

**Theorem** **3.** *Let the assumptions of Theorem 1 hold, $f_{i}\equiv 0$ for all i=1,2 and the initial data $\left(u_{0},b_{0}\right)\in B_{R}\left(0\right),$ where R satisfies *(16)*. Then, the estimate*

(78) $$\begin{array}{c} E\left(k,t\right)\leq 4\pi R_{1}^{2}, \end{array}$$

*holds for all k and all t, where $R_{1}$ is as in Theorem 1. Moreover, when $f_{i}≢0$ for some i=1,2, *(78)* still holds with $R_{1}$ replaced by $R_{1}\left(t\right)$ which is still finite and possibly grows in time.*

**Proof of Theorem** **3.** When $f_{i}\equiv 0,$ we have from (77) and Theorem 1 that

$$\begin{array}{ccc} E(k,t) & = & \int_{|\xi |=k}\left(\right|\hat{u}{\left(\xi ,t\right)|}^{2}+|\hat{b}\left(\xi ,t\right){|}^{2})dS\left(\xi \right)\\ & \leq & \int_{|\xi |=k}\frac{R_{1}^{2}}{k^{2}}dS\left(\xi \right)\\ & = & 4\pi R_{1}^{2}. \end{array}$$

Here we used the fact that the surface area of a sphere with radius *k* is equal to $4\pi k^{2}$.

When the external forces on the system, $f_{i}≢0,$ for some i=1,2 the proof above remains same with $R_{1}$ replaced with $R_{1}\left(t\right)$. With this we complete the proof. □

**Theorem** **4.** *Suppose the initial data $\left(u_{0},\;b_{0}\right)\in B_{R}\left(0\right),$ where R satisfy the conditions of Theorem 3 and the forces $f_{i}\in L_{loc}^{\infty }\left(\left[0,\infty \right];H^{-1}\left(D\right)\cap L^{2}\left(D\right)\right)$ for i=1,2 is bounded as it appears in *(50)*. Then for every T, we have*

(79) $$\begin{array}{c} \frac{1}{T}\int_{0}^{T}E\left(k,t\right)dt\leq \frac{4\pi R_{2}^{2}\left(T\right)}{\min\left(\nu ,\eta \right)Tk^{2}}, \end{array}$$

*where $R_{2}$ is as in Theorem 2.*

**Proof of Theorem** **4.** From (77) we have,

$$\begin{array}{ccc} \frac{1}{T}\int_{0}^{T}E\left(k,t\right)dt & = & \frac{1}{T}\int_{0}^{T}\int_{|\xi |=k}\left(\right|\hat{u}{\left(\xi ,t\right)|}^{2}+|\hat{b}\left(\xi ,t\right){|}^{2})dS\left(\xi \right)dt\\ & = & \frac{1}{T}\int_{0}^{T}\int_{|\xi |=k}\left(\right|{\hat{\chi }}_{k}\left(\xi \right)\hat{u}{\left(\xi ,t\right)|}^{2}+|{\hat{\chi }}_{k}\left(\xi \right)\hat{b}\left(\xi ,t\right){|}^{2})dS\left(\xi \right)dt\\ & \leq & \int_{|\xi |=k}\frac{1}{T}\frac{R_{2}^{2}\left(T\right)}{\min\left(\nu ,\eta \right)k^{4}}dS\left(\xi \right)\;\\ & \leq & 4\pi k^{2}\frac{R_{2}^{2}\left(T\right)}{\min\left(\nu ,\eta \right)Tk^{4}}=\frac{4\pi R_{2}^{2}\left(T\right)}{\min\left(\nu ,\eta \right)Tk^{2}}. \end{array}$$

Here we used (49) and the proof is complete. □

**Remark** **4.** *Theorem 3 and *(15)* imply that the bound of E(k,t) is fully determined by the initial data $\left(u_{0},b_{0}\right)$ and the nature of the non-homogeneous external forces $f_{1}$ and $f_{2}$. Additionally, when no external force is applied to the system, the spectral energy remains uniformly bounded through out the entire process.*

**Theorem** **5.** *Let the assumptions of Theorems 3 and 4 hold. Then the following are true about the inertial range of *(2)*:*

*1.*  *Inequality *(80)* is a necessary condition on the parameters so that E(k,t) exhibits K-41-like phenomenon.*
(80) $$\begin{array}{c} {\left(\min(\nu ,\eta )\right)}^{5/6}C_{0}\epsilon^{2/3}\leq 4\pi {\left(\frac{R_{2}\left(T\right)}{\sqrt{T}}\right)}^{5/3}R_{1}^{\frac{1}{3}}\left(T\right). \end{array}$$
*2.*  *An absolute lower bound for the inertial range is given by*
(81) $$\begin{array}{c} k_{1}=\frac{C_{0}^{3/5}\epsilon^{2/5}}{{\left(4\pi R_{1}^{2}\right)}^{3/5}}. \end{array}$$
*3.*  *An absolute upper bound for the inertial range is given by*
(82) $$\begin{array}{c} k_{2}={\left(\frac{4\pi }{C_{0}\min\left(\nu ,\eta \right)}\right)}^{3}\frac{1}{\epsilon^{2}}\frac{R_{2}^{6}\left(T\right)}{T^{3}}. \end{array}$$

**Proof of Theorem** **5.** Define set *S* by

(83) $$\begin{array}{c} S:=\left\{\left(k,E\right):0\leq E\left(k,\cdot \right)\leq 4\pi R_{1}^{2},\;0\leq E\left(k,\cdot \right)\leq \frac{4\pi R_{2}^{2}}{\min\left(\nu ,\eta \right)Tk^{2}}\right\}. \end{array}$$

**Figure 1:**
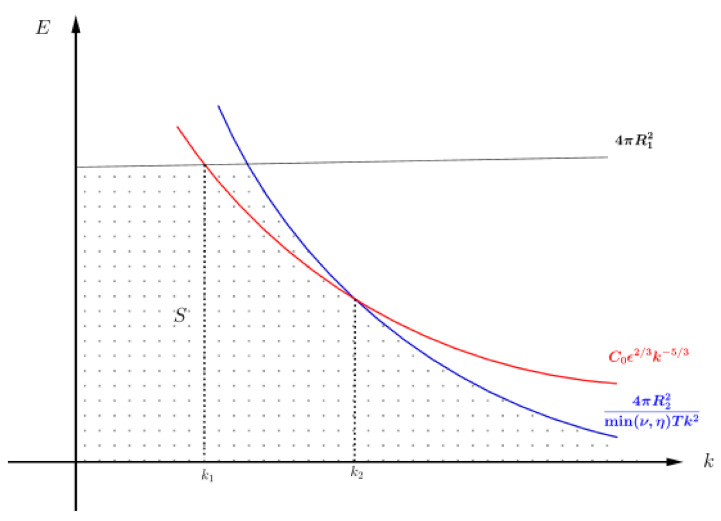
Sketch of region *S* and set *A* when condition (84) is satisfied.

Let

$$\begin{array}{c} A:=\{\left(k,E\right):E=E_{K}\left(k\right)\}\cap S, \end{array}$$

be part of the graph of $E_{K}\left(k\right)$ that lies in region *S*. Figure 1 shows how sets *S* and *A* are related.

Due to Theorem 3 we know that the spectral energy of our system is bounded from above by $4\pi R_{1}^{2}$ when $f_{i}\equiv 0$ for all i=1,2 or $4\pi R_{1}^{2}\left(T\right)f_{i}≢0$ for some i=1,2. Furthermore, from Theorem 4 the time average is bounded by

$$\begin{array}{c} \frac{4\pi R_{2}^{2}\left(T\right)}{\min\left(\nu ,\eta \right)Tk^{2}}. \end{array}$$

Thus, set *S* represents the behavior of the function E(k,t), and set *A* is a set where E(k,t) behaves accordingly with K-41. Therefore, if A=∅ then E(k,t) does not exhibit K-41-like phenomenon.

Note that for *A* to be non-empty the point where graphs of $E_{K}\left(k\right)$ and $\frac{4\pi R_{2}^{2}}{\min\left(\nu ,\eta \right)Tk^{2}}$ must intersect below the line $E=4\pi R_{1}^{2}$, as in Figure 1, and the intersection occurs when

$$\begin{array}{ccc} C_{0}\epsilon^{2/3}k^{-5/3} & = & \frac{4\pi R_{2}^{2}\left(T\right)}{\min\left(\nu ,\eta \right)Tk^{2}}\\ \Rightarrow k & = & {\left(\frac{4\pi R_{2}^{2}\left(T\right)}{\min\left(\nu ,\eta \right)TC_{0}\epsilon^{2/3}}\right)}^{3}. \end{array}$$

Moreover, the graph of $E_{K}\left(k\right)$ intersects the line $E=4\pi R_{1}^{2}$ below the graph of $\frac{4\pi R_{2}^{2}}{\min\left(\nu ,\eta \right)Tk^{2}}$, as in Figure 1, which occurs when

$$\begin{array}{ccc} C_{0}\epsilon^{2/3}k^{-5/3} & = & 4\pi R_{1}^{2}\\ \Rightarrow k & = & {\left(\frac{4\pi R_{1}^{2}}{C_{0}\epsilon^{\frac{2}{3}}}\right)}^{\frac{-3}{5}}. \end{array}$$

Therefore, $E_{K}\left(k\right)$ enters region *S* at $k={\left(\frac{4\pi R_{1}^{2}}{C_{0}\epsilon^{\frac{2}{3}}}\right)}^{\frac{-3}{5}}$ and leaves at $k={\left(\frac{4\pi R_{2}^{2}\left(T\right)}{\min\left(\nu ,\eta \right)TC_{0}\epsilon^{\frac{2}{3}}}\right)}^{3}$.

Now set,

$$\begin{array}{c} k_{1}={\left(\frac{4\pi R_{1}^{2}}{C_{0}\epsilon^{2/3}}\right)}^{-3/5},\;k_{2}={\left(\frac{4\pi R_{2}^{2}\left(T\right)}{\min\left(\nu ,\eta \right)TC_{0}\epsilon^{2/3}}\right)}^{3}, \end{array}$$

where $k_{1}$ is the intersection of the graphs of $E_{K}\left(k\right)$ and the constant function $4\pi R_{1}^{2}$ and $k_{2}$ is the intersection of $E_{K}\left(k\right)$ and $\frac{4\pi R_{2}^{2}\left(T\right)}{\min\left(\nu ,\eta \right)Tk^{2}}$. Thus the portion of the graph of $E_{K}\left(k\right)$ remains in region *S* as long as *k* is between $k_{1}$ and $k_{2}$ and $k_{1}\leq k_{2}$, see Figure 1.

**Figure 2:**
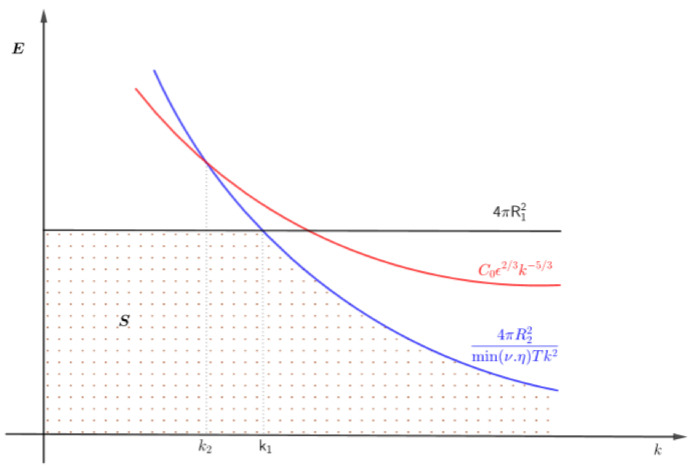
Sketch of region *S* and set *A* when condition (84) is not satisfied.

Observe from Figure 2 that if we push the graph of $\frac{4\pi R_{2}^{2}\left(T\right)}{\min\left(\nu ,\eta \right)Tk^{2}}$ to the left so that it intersects $E_{K}\left(k\right)$ above the graph of $4\pi R_{1}^{2}$, then we get $k_{1}>k_{2}$ and the graph of $E_{K}\left(k\right)$ will not pass through region *S* which in turn gives A=∅. Hence, *A* remains non-empty only when $k\in [k_{1},k_{2}]$.

Therefore, for the flow model (76) exhibit K-41-like MHD phenomenon we need the necessary condition

$$\begin{array}{cc} {\left(\frac{4\pi R_{1}^{2}}{C_{0}\epsilon^{2/3}}\right)}^{-3/5} & \leq {\left(\frac{4\pi R_{2}^{2}\left(T\right)}{\min\left(\nu ,\eta \right)TC_{0}\epsilon^{2/3}}\right)}^{3}, \end{array}$$

to be satisfied. Hence,

(84) $$\begin{array}{ccc} C_{0}\min{\left(\nu ,\eta \right)}^{5/6}\epsilon^{2/3} & \leq & 4\pi {\left(\frac{R_{2}\left(T\right)}{\sqrt{T}}\right)}^{5/3}R_{1}^{\frac{1}{3}}\left(T\right). \end{array}$$

This completes the proof Theorem 5. □

## 4. Conclusions

In this work, we have investigated the Leray weak solution of the deterministic MHD model (2) for the K-41-like MHD phenomenon in the presence and absence of external forces. In the process it is shown in Section 2.2 that when the external the solution field (u,b) is bounded in the Fourier space (Theorems 1 and 2) and the bound depends on the data. When the external forces $f_{1}$ and $f_{2}$ are identically 0, the bound is uniform. It is also shown that the spectral energy of the system E(k,t) is bounded, and when the external forces $f_{i}\equiv 0$ for i=1,2 the bound is uniform (Theorem 3) and the average in time decreases in time and decays proportional to $k^{-2}$. When $f_{i}≢0$ for some i=1,2 the bonds of E(k,t) possibly depend on time. The other important result of this work is the explicit formulation of the inertial range bounds and setting the necessary condition on the parameters for the model to behave accordingly with K-41 (Theorem 5). The lower bound

$$\begin{array}{c} k_{1}={\left(\frac{4\pi R_{1}^{2}}{C_{0}\epsilon^{2/3}}\right)}^{-3/5}, \end{array}$$

is a constant in time when $f_{i}\equiv 0$ for i=1,2 and possibly decreases in time when $f_{i}≢0$ for some i=1,2. The upper bound of the inertial range

$$k_{2}={\left(\frac{4\pi R_{2}^{2}\left(T\right)}{\min\left(\nu ,\eta \right)TC_{0}\epsilon^{2/3}}\right)}^{3},$$

decreases in time when $f_{i}\equiv 0$ for i=1,2 and will remain decreasing as long as the $R_{2}\propto T^{\alpha }$ and α<1/2. For the case where $f_{i}\equiv 0$ for i=1,2, $R_{1}$ and $R_{2}$ are constants independent of time and at time $T=T_{0}$, where

(85) $$\begin{array}{c} T_{0}:=\frac{{\left(4\pi \right)}^{6/5}R_{2}^{2}R_{1}^{2/5}}{\epsilon^{4/5}C_{0}^{6/5}\min\left(\nu ,\eta \right)} \end{array}$$

we get $k_{1}=k_{2}$. This means that for any time $T>T_{0}$, the spectral range is empty. Consequently, time $T_{0}$ appears to be the maximal time to exhibit K-41 in the system.

If we assume that the dissipation rate is time dependent then (85) gives

(86) $$\begin{array}{c} \epsilon \left(T_{0}\right)=\frac{{\left(4\pi \right)}^{3/2}R_{1}^{1/2}R_{2}^{5/2}}{T_{0}^{5/4}\min{\left(\nu ,\eta \right)}^{5/4}C_{0}^{3/2}}. \end{array}$$

The time $T_{0}$, being the maximal time (86), must be the minimum dissipation rate to maintain a spectral behavior.

## Data Availability

Not applicable.
